# Real-world deep brain stimulation programming practices for Parkinson’s disease in India: variability, technology adoption, and emerging trends from a multicentre clinician survey across India

**DOI:** 10.3389/fneur.2026.1892612

**Published:** 2026-08-31

**Authors:** Pramod Kumar Pal, Roopa Rajan, Syam Krishnan Nair, Ravi Yadav, Mohit Anand, Shankar Balakrishnan, Manish Baldia, Anirban Deep Banarjee, Rupam Borgohain, Jacky Ganguly, Dhanunjay Rao Ginjupally, Vikram V Holla, Guruprasad Hosurkar, Nitesh Kamble, Rukmini Mridula Kandadai, Akshat Kayal, Abhijeet Kumar Kohat, Hrishikesh Kumar, Rohan R Mahale, Vaibhav Matur, Sreeram Prasad, R. Shiva Kumar, Atif Sheikh, Kuldeep Shetty, Sumit Singh, Anil Venkatachalam, Vijay Shankar, Kavadisseril Vivekanandan Vysakh, Sireesha Yareeda

**Affiliations:** 1 Department of Neurology, National Institute of Mental Health and Neurosciences (NIMHANS), Bangalore, India; 2 Department of Neurology, All India Institute of Medical Sciences (AIIMS), Delhi, India; 3 Department of Comprehensive Care Centre for Movement Disorders, Sree Chitra Tirunal Institute for Medical Sciences and Technology (SCTIMST), Trivandrum, Kerala, India; 4 Department of Neurology, Artemis Hospital, Gurgaon, India; 5 Department of Neurology, Rela Hospital, Chennai, India; 6 Department of Neurosurgery, Wockhardt Hospital, Mumbai, India; 7 Department of Neurosurgery, Medanta Institute of Neurosciences, Gurgaon, India; 8 Department of Parkinson’s and Movement Disorders Research Centre (PDMDRC), Yashoda Hospitals, Hyderabad, India; 9 Department of Neurology, Movement Disorder Centre, Institute of Neurosciences Kolkata, India; 10 Department of Neurosurgery, Apollo Health City, Hyderabad, India; 11 Department of Neurology, Head of Parkinson’s Disease and Movement Disorder Programme, Krishna Institute of Medical Sciences (KIMS) Hospitals, Bangalore, India; 12 Department of Neurosurgery Kokilaben Dhirubhai Ambani Hospital, Mumbai, India; 13 Department of Neurology, Dau Kalyan Singh Post Graduate Institute and Research Centre, Raipur, India; 14 Department of Neurology and Neurosurgery, Narayana Multispeciality Hospital, Jaipur, India; 15 Advanced Center for Movement Disorders and DBS, Rajagiri Hospital, Kochi, Kerala, India; 16 Department of Neurology, Manipal Hospital, Bangalore, India; 17 Department of Neurology, Christian Medical College, Vellore, Tamil Nadu, India; 18 Department of Neurology, NH Mazumdar Shaw Medical Centre, Bangalore, India; 19 Department of Neurology, Apollo Hospitals, Navi Mumbai, India; 20 Department of Neurology, Apollo Hospitals, Chennai, India; 21 Department of Neurology, Caritas Neuro Sciences, Kottayam, India; 22 Department of Neurology, Nizam’s Institute of Medical Sciences (NIMS), Hyderabad, India

**Keywords:** DBS programming, deep brain stimulation, Parkinson’s disease, real-world evidence, sensing-enabled DBS

## Abstract

**Introduction:**

Deep brain stimulation (DBS) outcomes in Parkinson’s disease (PD) are strongly influenced by postoperative programming, yet real-world workflows and adoption of newer technologies, including directional stimulation, sensing-enabled systems, and adaptive DBS, vary across clinical settings. This study aimed to describe contemporary real-world DBS programming practices for PD among clinicians across India.

**Methods:**

We conducted an anonymous, cross-sectional, web-based survey of Indian clinicians actively programming DBS for PD with ≥1 year of experience. The survey assessed initial programming workflows, symptom-specific optimization strategies, medication adjustment practices, use of sensing-enabled technologies, approaches to suboptimal response, respondent-level workflow patterns, and perceptions of adaptive DBS. Results were analyzed descriptively using item-specific denominators; multi-select responses were summarized as option-level proportions.

**Results:**

Fifty of 54 approached clinicians completed the survey. Respondents represented 37 DBS centres across 18 Indian cities. The subthalamic nucleus was the most commonly selected target (42/50, 84.0%). Stimulation was most often initiated within 1–2 weeks (25/50, 50.0%) or 3–4 weeks (14/50, 28.0%) after implantable pulse generator implantation. Initial programming strategies were captured as a multi-select item; the most frequently selected strategy was a combination of monopolar review and BrainSense (35/50, 70.0%), followed by traditional monopolar review (22/50, 44.0%). Current BrainSense use was reported by 42/50 respondents (84.0%); among current users, initiation most often occurred at initial programming (26/42, 61.9%). Symptom-specific optimization strategies varied by clinical problem: amplitude adjustment was most frequently selected for bradykinesia (42/50, 84.0%) and rigidity (45/50, 90.0%), while frequency adjustment was most frequently selected for tremor (47/50, 94.0%) and freezing of gait (43/50, 86.0%). For suboptimal response, imaging-based lead localization strategies were frequently selected, particularly pre−/post-operative imaging merge with lead location review (44/50, 88.0%). Exploratory respondent-level analysis identified overlapping workflow domains, including sensing-integrated programming (26/50, 52.0%) and technology-assisted troubleshooting (29/50, 58.0%); 31/50 respondents (62.0%) fell into the high workflow/technology-integration score category. Most respondents intended to incorporate adaptive DBS into routine clinical practice (45/50, 90.0%).

**Conclusion:**

This multicentre survey provides a snapshot of contemporary DBS programming practices for PD in India, highlighting practice variability, increasing adoption of technology-assisted programming, and strong interest in adaptive DBS. These findings support the need for context-specific training and future outcome-based research.

## 1 Introduction

Deep brain stimulation (DBS) is an established therapy for people with Parkinson’s disease (PD) who experience medication-refractory tremor, disabling motor fluctuations, and/or dyskinesias despite optimized pharmacotherapy (1).

However, long-term benefit from DBS is not driven by surgery alone, post-operative programming is a critical determinant of clinical outcomes. DBS programming is inherently iterative, requiring repeated adjustments of stimulation parameters and contact configurations to balance symptom control against stimulation-related adverse effects. Given the large number of possible parameter combinations, real-world programming often relies on clinician experience, local workflows, and available technology rather than strictly defined algorithms (2).

DBS technology has evolved considerably over the past decade. Directional leads and advanced programming features enable current steering and field shaping, while sensing-enabled platforms provide tools to support programming and longitudinal optimisation (3, 4). Adaptive or closed-loop DBS has further emerged as a step toward personalised neuromodulation, with recent trials, including ADAPT-PD, demonstrating feasibility of adaptive algorithms in selected patients previously stable on continuous stimulation (5). However, integration of these technologies into routine practice remains variable and is influenced by training exposure, interpretability, time constraints, and perceived clinical utility.

Despite expert consensus statements and commonly used institutional frameworks, high-level evidence defining standardized DBS programming strategies for PD remains limited. Consequently, substantial variability persists across clinicians and centres, particularly in initial programming workflows, symptom-specific optimisation strategies, medication adjustment timing, and approaches to suboptimal response. International surveys of DBS experts have demonstrated near-consensus in certain domains such as the central role of monopolar review and amplitude titration alongside marked heterogeneity in others, highlighting the importance of systematically describing real-world practice patterns (6, 7).

In India, access to DBS is expanding across heterogeneous practice environments with diverse multidisciplinary models and resource constraints yet published data describing real-world DBS programming practices remain limited. To address this gap, we conducted a structured survey of experienced DBS programmers in India to descriptively characterise contemporary programming practices across key domains. The aim was to document current practice variability and adoption patterns without proposing prescriptive recommendations or inferring superiority of any specific approach.

## 2 Materials and methods

### 2.1 Study design

We conducted a cross-sectional, anonymous, web-based survey to describe real-world DBS programming practices for PD in India. The objective was to characterise contemporary workflows across the DBS care pathway, including initial programming, subsequent optimisation, symptom-specific parameter adjustments, use of sensing-enabled features, medication adjustment practices, and approaches to suboptimal outcomes. The study was descriptive in intent and was not designed to evaluate clinical outcomes or determine superiority of any programming strategy. Survey domains and wording were informed by previously published expert surveys (6, 7) on DBS programming variability, adapted to the Indian clinical context and to the technologies and workflows addressed in the questionnaire.

### 2.2 Setting and participants

The survey was administered using Microsoft Forms. The survey link was sent to professional email addresses of eligible clinicians between September 2025 and December 2025. Responses were collected electronically and exported for analysis. Eligible participants were clinicians actively performing DBS programming for PD with a minimum of one year of programming experience at the time of participation. Of 54 clinicians approached, 50 completed the survey. The respondents did not represent 50 unique DBS centres in several high-volume centres, multiple clinicians share programming responsibilities, and more than one programmer from such centres was intentionally included to capture programmer-level real-world practices.

Respondents represented 37 DBS centres across 18 Indian cities. Most centres contributed one respondent (30/37, 81.1%), while five centres contributed two respondents (13.5%) and two centres contributed three respondents (5.4%). The survey was geographically distributed across India but was based on purposive clinician sampling and was not designed to provide a nationally representative estimate of all DBS centres or programmers.

### 2.3 Questionnaire development and measures

The questionnaire included single-choice, multiple-choice, ranking, and multi-select items. Multi-select formats were used for domains such as symptom optimisation strategies, patient education, medication-reduction decision factors, BrainSense survey purpose, BrainSense feature use, and approaches to suboptimal outcomes. For all multi-select items, percentages are reported as the proportion of respondents selecting each option, and therefore totals may exceed 100%. Item-specific denominators are stated throughout where non-response or subgroup restriction applied. The questionnaire has been attached as Supplementary material 1.

### 2.4 Bias and limitations

Sampling was purposive and focused on experienced DBS programmers, which may over-represent clinicians with higher DBS engagement or access to specific technologies. Inclusion of multiple respondents from some centres may result in centre-level clustering; accordingly, the unit of analysis was the individual clinician. All responses were self-reported and could not be independently verified, with potential for recall or interpretation bias. Survey anonymity and non-evaluative framing were used to mitigate reporting bias.

### 2.5 Analysis

Categorical variables were summarised as counts and percentages using item-specific denominators. Single-choice items were reported as mutually exclusive categories. For multi-select items, each response option was tabulated separately, and percentages reflect the proportion selecting that option rather than summing to 100%. Residual categories such as ‘other’, ‘plan to use’, ‘not aware’, or non-response were retained where applicable to preserve denominator transparency. An exploratory, non-causal subgroup comparison examined timing of BrainSense initiation (early vs. late/conditional) and perceived maturity of advanced programming tool adoption (OptiStim/ShapeLock).

We also performed an exploratory respondent-level workflow analysis to complement item-level descriptive reporting. Because the sample size was modest and several questionnaire domains were multi-select or had mixed response structures, formal clustering was not attempted. Instead, we used prespecified, transparent, non-mutually exclusive workflow domains reflecting (1): clinically anchored conventional programming (2), sensing-integrated programming (3), technology-assisted troubleshooting, and (4) structured follow-up-oriented workflow. We also derived an exploratory workflow/technology-integration score (range 0–9) by summing prespecified respondent-level indicators of early sensing use, survey-based integration, advanced troubleshooting, and structured follow-up. These analyses were exploratory and hypothesis-generating only.

### 2.6 Reporting and ethics

This study is reported in accordance with STROBE guidelines for web-based cross-sectional surveys. Ethics committee/IRB approval was not required as the study involved an anonymous clinician survey with no patient-level data, intervention, or more than minimal risk.

## 3 Results

Fifty clinicians completed the survey (50/54 approached). All respondents met the eligibility criterion of at least one year of DBS programming experience in PD. The survey included clinicians from 37 hospitals across 18 cities representing the North, South, East and West regions of India. Some high-volume centres contributed more than one respondent, reflecting shared DBS programming responsibilities within those institutions.

### 3.1 Respondent and practice characteristics

Practice setting was reported by all respondents (single-choice, *n* = 50). Most worked in corporate hospitals (32/50, 64.0%), followed by government hospitals (8/50, 16.0%), private medical colleges (6/50, 12.0%), and non-corporate or standalone hospitals (4/50, 8.0%).

The 50 respondents represented 37 DBS centres across 18 cities. Thirty centres contributed one respondent (30/37, 81.1%), five centres contributed two respondents (5/37, 13.5%), and two centres contributed three respondents (2/37, 5.4%); the maximum number of respondents from any single centre was three. Regional distribution included South India (35/50, 70.0%), West India (6/50, 12.0%), North India (5/50, 10.0%), and East India (4/50, 8.0%) (Table 1).

**Table 1:** Geographic distribution of respondents and participating DBS centres across India; data are presented as *n* (%).

Characteristic	Value
Total respondents	50
Total participating centres	37
Total cities represented	18
Centres contributing one respondent	30 (81.1%)
Centres contributing two respondents	5 (13.5%)
Centres contributing three respondents	2 (5.4%)
Maximum respondents from a single centre	3
South India	35 respondents (70.0%)
West India	6 respondents (12.0%)
North India	5 respondents (10.0%)
East India	4 respondents (8.0%)

Monthly PD clinic volume was reported by all respondents (single-choice, *n* = 50). Respondents were distributed across 1–15 patients/month (14/50, 28.0%), 16–30/month (9/50, 18.0%), 31–50/month (14/50, 28.0%), and >50/month (13/50, 26.0%).

The number of new PD patients seen per month for initial DBS programming was also reported by all respondents (single-choice, *n* = 50), with the majority reporting 1–5 patients/month (39/50, 78.0%), followed by 6–10/month (8/50, 16.0%), 11–15/month (2/50, 4.0%), and >15/month (1/50, 2.0%) (Supplementary Tables 1–3).

### 3.2 Initial DBS programming session

Target preference was reported by all respondents as a single-choice item (*n* = 50). The subthalamic nucleus (STN) was most frequently selected (42/50, 84.0%), followed by globus pallidus internus (GPi) (7/50, 14.0%) and other targets (1/50, 2.0%) (Supplementary Table 4).

Timing of stimulation initiation after implantable pulse generator (IPG) implantation was also reported by all respondents (single-choice, *n* = 50). Most initiated stimulation within 1–2 weeks (25/50, 50.0%) or 3–4 weeks (14/50, 28.0%) after implantation, while smaller proportions reported initiation at 1–3 months (3/50, 6.0%), >3 months (1/50, 2.0%), or other time points (7/50, 14.0%) (Supplementary Table 5).

The clinician performing initial programming was reported by all respondents (*n* = 50). Movement disorder consultants were most commonly involved (39/50, 78.0%), followed by movement disorder fellows (9/50, 18.0%) and neurosurgeons (9/50, 18.0%) (Supplementary Table 6).

Time allocated for programming a patient with bilateral leads was reported by all respondents (single-choice, *n* = 50) and was most often 30–60 min (21/50, 42.0%), followed by 61–90 min (19/50, 38.0%), 91–120 min (6/50, 12.0%), and >120 min (4/50, 8.0%) (Supplementary Table 7).

Preference for ring versus segmented stimulation at initiation was also reported by all respondents (single-choice, *n* = 50). Ring electrodes were most frequently selected (30/50, 60.0%), followed by ring or segmented electrodes depending on patient presentation (14/50, 28.0%) and segmented electrodes (6/50, 12.0%) (Supplementary Table 8).

### 3.3 Initial programming strategy and advanced features

**Figure 1:**
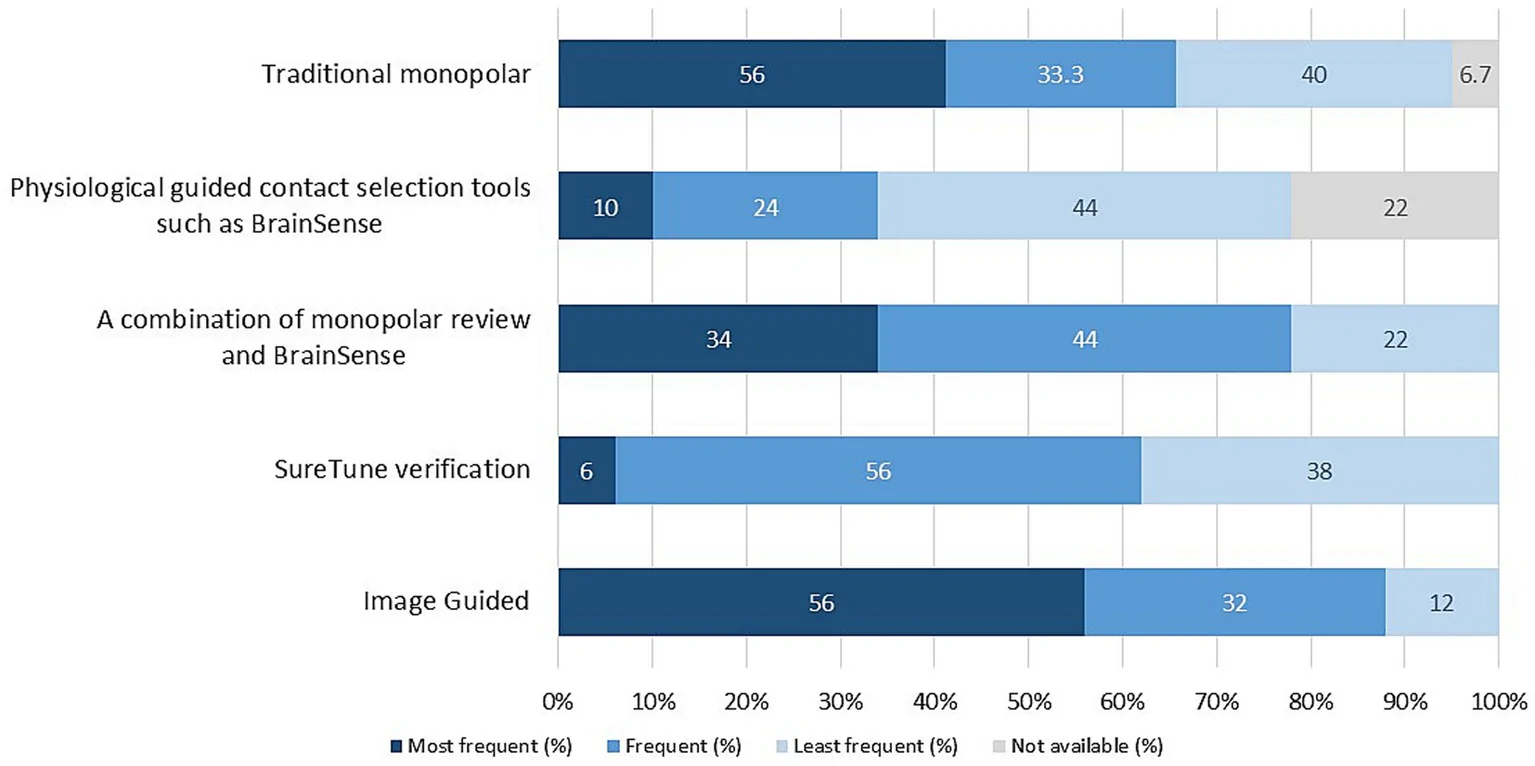
Participant ranking for the commonly used initial programming strategy (*N* = 50). Respondents (*n* = 50) ranked the frequency of use of initial programming strategies as most frequent, frequent, least frequent, or not available. Bars represent the percentage of respondents selecting each category for each strategy. Traditional monopolar and image-guided programming were most commonly ranked as the most frequently used strategies (56% each), while SureTune verification was predominantly ranked as frequent (56%) and physiologically guided contact selection tools such as BrainSense were more often ranked as least frequent (44%) or not available (22%). Percentages may not total exactly 100% because of rounding.

Usual initial programming strategies were captured as a multi-select item and were reported by all respondents (*n* = 50). The most frequently selected strategy was a combination of monopolar review and BrainSense (35/50, 70.0%), followed by traditional monopolar review (22/50, 44.0%), SureTune verification (9/50, 18.0%), physiologically guided contact selection tools such as BrainSense (8/50, 16.0%), and other approaches (2/50, 4.0%). Because multiple responses were permitted, percentages for this item do not sum to 100% (Supplementary Table 9). Respondent-reported rankings of the frequency of use of initial programming strategies are shown in Figure 1 and Supplementary Table 10. Traditional monopolar programming and image-guided programming were most commonly ranked as the most frequently used strategies (56% each), followed by a combination of monopolar review and BrainSense (34%). SureTune verification was most commonly rated as a frequent strategy (56%), whereas physiologically guided contact selection tools such as BrainSense were more often ranked as least frequent (44%) or reported as unavailable (22%).

**Figure 2:**
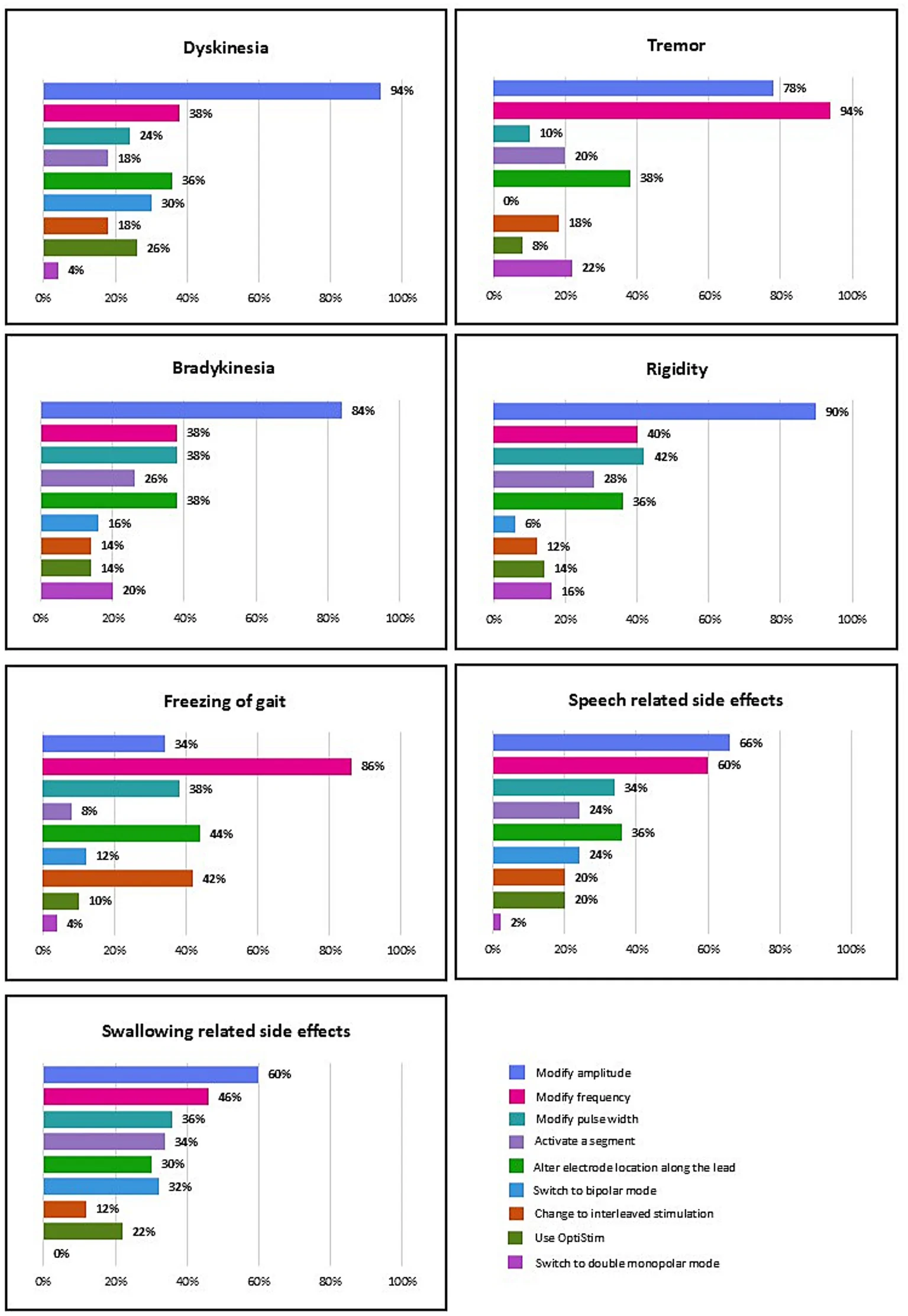
Schematic overview of the DBS programming workflow. Following clinical assessment and initial activation of the DBS system, a monopolar review is performed to identify the optimal stimulation contact(s). Stimulation parameters (amplitude, pulse width, and frequency) are then optimized, with adjunctive tools such as BrainSense, imaging-based lead review, and software-assisted programming platforms (e.g., SureTune/OptiStim) used when appropriate. Clinical response is subsequently assessed, and programming is iteratively refined to maximize symptom control while minimizing adverse effects.

Familiarity with OptiStim/ShapeLock was reported as a single-choice item by all respondents (*n* = 50). Nineteen respondents (19/50, 38.0%) reported the feature as valuable for tailoring therapy, 17/50 (34.0%) reported potential utility but indicated the need for additional training, and 14/50 (28.0%) reported not being familiar with the feature (Supplementary Table 11). A schematic overview of the DBS programming strategy has been represented in Figure 2.

### 3.4 Symptom-specific optimization strategies

All symptom-specific optimisation items were asked as multi-select questions (select up to 3 options), and all 50 respondents contributed to each symptom domain (*n* = 50 per symptom). Accordingly, percentages represent the proportion of respondents selecting a given strategy for that symptom and are not mutually exclusive.

Across symptoms, amplitude adjustment was frequently selected, whereas tremor and freezing of gait showed greater emphasis on frequency-based strategies. For dyskinesia, amplitude adjustment was selected by 47/50 respondents (94.0%), followed by frequency changes (19/50, 38.0%) and alterations in electrode location along the lead (18/50, 36.0%).

For tremor, frequency was selected by 47/50 respondents (94.0%) and amplitude by 39/50 (78.0%). For bradykinesia, amplitude was selected by 42/50 (84.0%), while for rigidity, amplitude was selected by 45/50 (90.0%).

**Figure 3:**
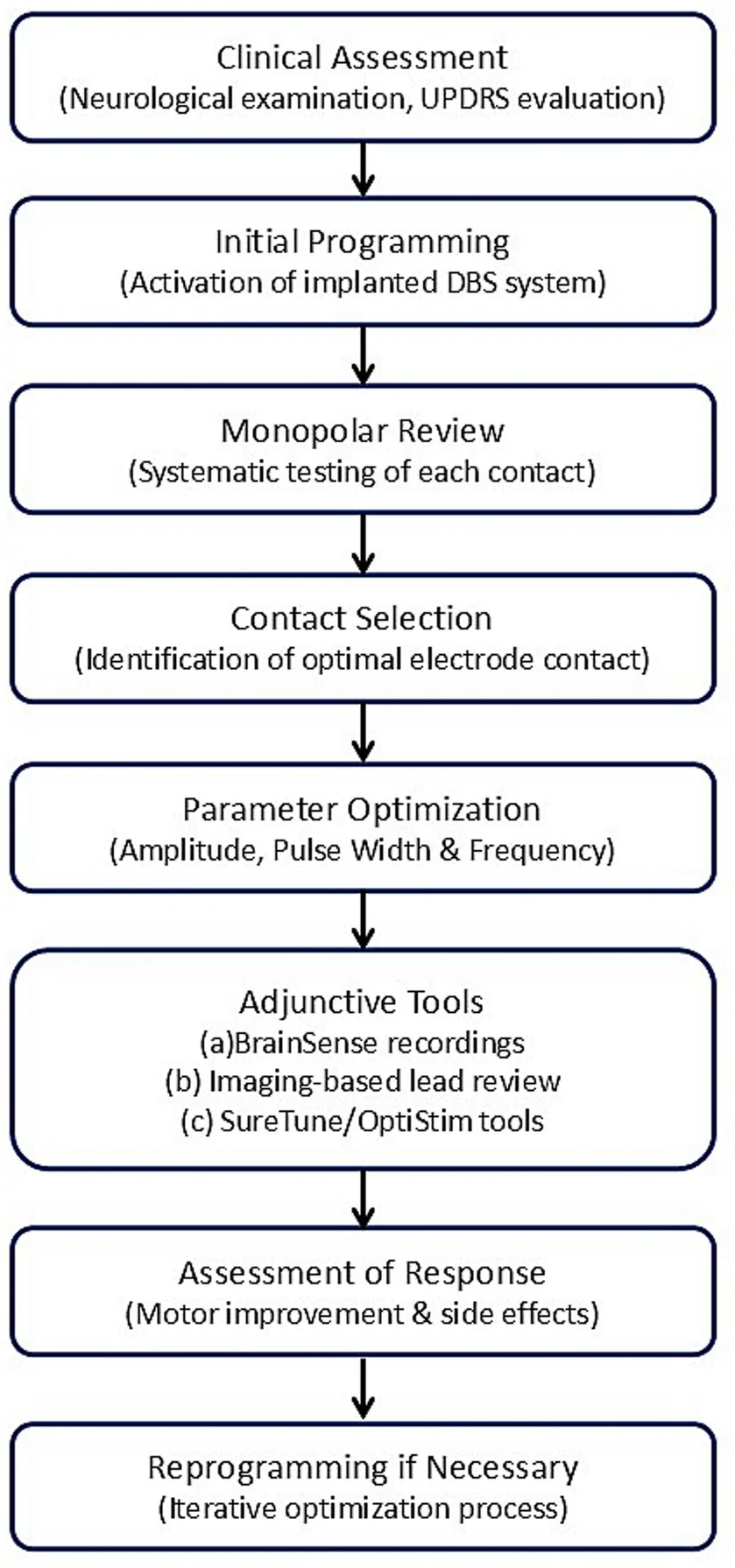
Symptom-specific DBS programming strategies (*N* = 50 per symptom domain). All symptom-specific optimisation items were asked as multi-select questions (select up to 3 options), and all 50 respondents contributed to each symptom domain. Percentages represent the proportion of respondents selecting a given strategy for each symptom and are not mutually exclusive. Across symptoms, amplitude adjustment was frequently selected, whereas tremor and freezing of gait showed greater emphasis on frequency-based strategies. Multi-select item (select up to 3 options); denominator for each symptom domai*n* = 50. Percentages represent option-level selection frequencies and may total >100%.

For freezing of gait, frequency was selected by 43/50 respondents (86.0%), followed by altered electrode location (22/50, 44.0%) and interleaving (21/50, 42.0%). Speech- and swallowing-related adverse effects were commonly managed using combinations of amplitude and frequency adjustment, with additional use of pulse-width and contact-level changes (Figure 3; Supplementary Tables 12–19).

### 3.5 Patient education, medication adjustment, and follow-up

**Figure 4:**
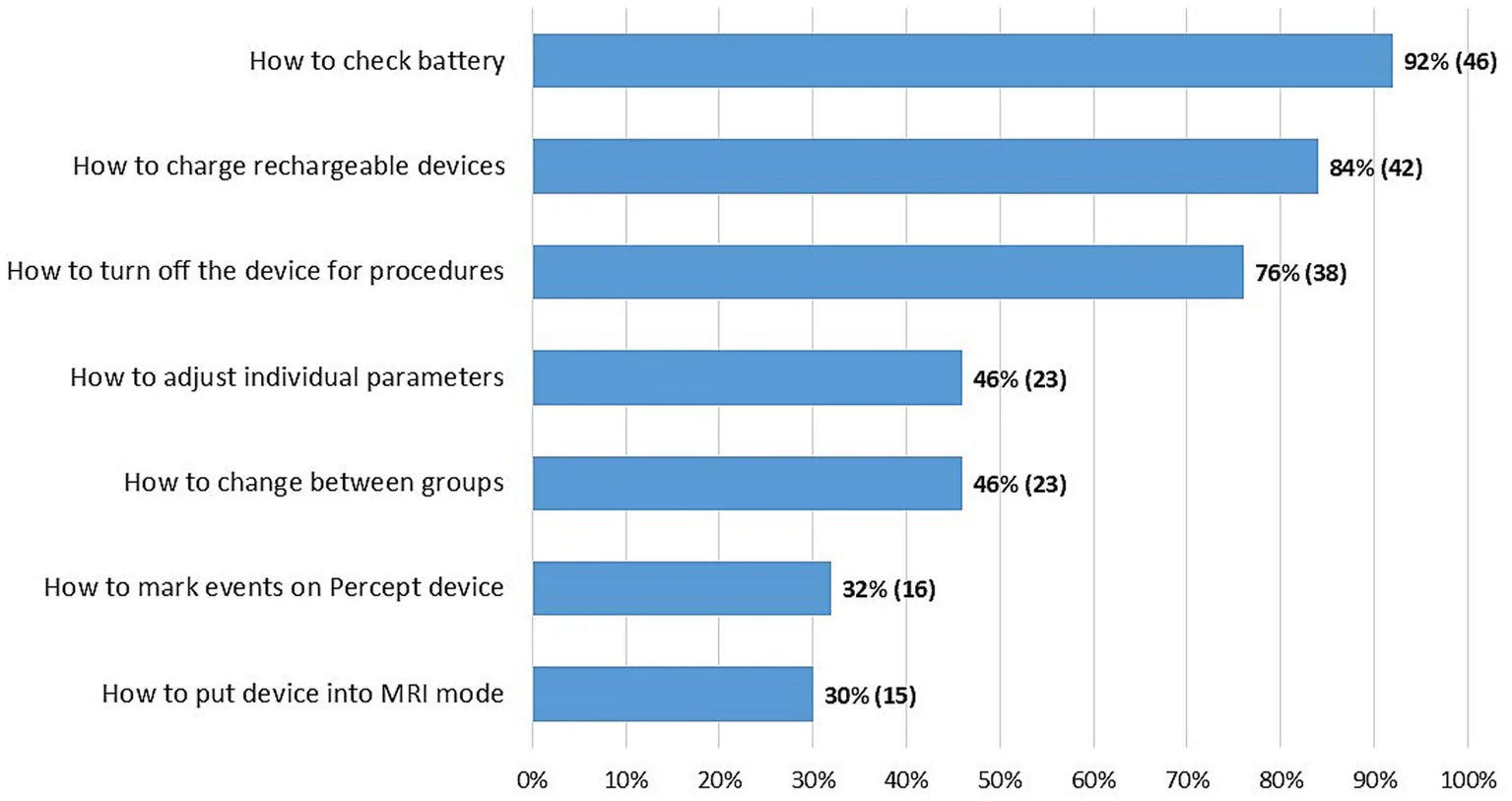
Patient education topics discussed at initial programming (*N* = 50). Patient education topics were captured as a multi-select item and reported by all respondents (*n* = 50). Bars represent the proportion of respondents selecting each education topic. The most frequently selected topics were battery checking (46/50, 92.0%), charging rechargeable devices (42/50, 84.0%), and turning off the device for procedures (38/50, 76.0%). Percentages reflect option-level selections and do not sum to 100%.

Patient education topics at initial programming were captured as a multi-select item and were reported by all respondents (*n* = 50); percentages therefore reflect option-level selections and do not sum to 100%. The most frequently selected topics were battery checking (46/50, 92.0%), charging rechargeable devices (42/50, 84.0%), and turning off the device for procedures (38/50, 76.0%). Parameter adjustment and changing between groups were each reported by 23/50 respondents (46.0%) (Figure 4; Supplementary Table 20).

Timing of medication reduction was assessed as a single-choice item for both STN and GPi programming (*n* = 50 for each target-related item). Following initial programming of DBS STN, medication reduction was most commonly reported at the time of initial programming (19/50, 38.0%) or one month after initial programming (12/50, 24.0%). Following initial programming of DBS GPi, the most common responses were no set time for medication reduction (16/50, 32.0%) and one month after initial programming (14/50, 28.0%) (Supplementary Tables 21, 22).

**Figure 5:**
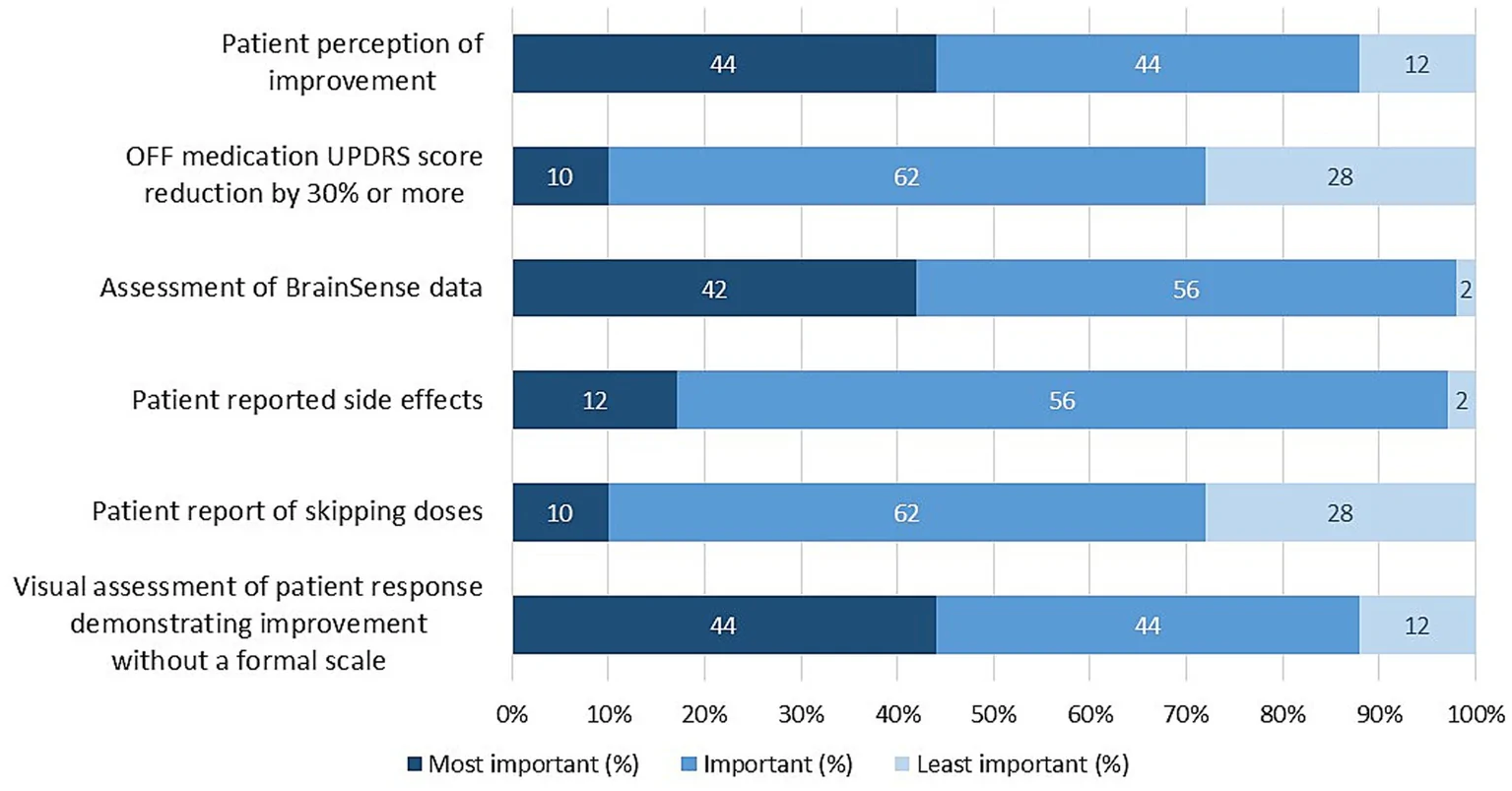
Importance rankings of factors used to guide medication reduction among all respondents (*N* = 50). Respondents (*n* = 50) ranked the importance of factors used to guide medication reduction as most important, important, or least important. Stacked bars represent the percentage of respondents assigning each importance level to a given factor. Assessment of BrainSense data (42%) and patient/clinician-perceived improvement (44% each) were most commonly ranked as the most important factors, while OFF-medication UPDRS score reduction ≥ 30% and patient reports of skipping doses were predominantly rated as important (62% each). Percentages may not total exactly 100% because of rounding.

Factors used to guide medication reduction were captured as a multi-select item and reported by all respondents (*n* = 50). The most frequently selected factors were patient-perceived improvement (34/50, 68.0%), OFF-medication UPDRS score reduction ≥30% (29/50, 58.0%), patient-reported side effects (22/50, 44.0%), visual assessment of patient response without a formal scale (21/50, 42.0%), and assessment of BrainSense data (15/50, 30.0%) (Supplementary Table 23). Respondent-reported rankings of the importance of these factors are presented in Figure 5 and Supplementary Table 24. Patient-perceived improvement and visual assessment of patient response were most frequently ranked as the most important factors (44% each), followed by assessment of BrainSense data (42%), whereas OFF-medication UPDRS score reduction ≥30% and patient reports of skipping doses were more commonly ranked as important (62% each).

Follow-up frequency after initial programming was assessed as a single-choice item in all respondents (*n* = 50) and was most often every month until stimulation was optimised (20/50, 40.0%) or every 2 weeks (19/50, 38.0%), with smaller proportions recommending every 2 months (5/50, 10.0%) or every 3 months (6/50, 12.0%). Asking patients to come off dopaminergic medications for subsequent programming sessions was also assessed in all respondents (single-choice, *n* = 50); 20/50 respondents (40.0%) reported doing so always, 17/50 (34.0%) almost always, 10/50 (20.0%) sometimes, and 3/50 (6.0%) never. Thus, 37/50 respondents (74.0%) reported doing so always or almost always (Supplementary Tables 25, 26).

### 3.6 BrainSense utilisation and adaptive DBS attitudes

Current BrainSense use was reported as a single-choice item by all respondents (*n* = 50): yes in 42/50 (84.0%), plan to use in 4/50 (8.0%), and no in 4/50 (8.0%). Subsequent BrainSense-related items were analysed using item-specific denominators restricted to current BrainSense users, as applicable.

Among BrainSense users, timing of initiation was available for *n* = 42 and occurred at initial programming in 26/42 (61.9%), after the initial programming session in 11/42 (26.2%), when clinical programming results were suboptimal in 3/42 (7.1%), and under other circumstances in 2/42 (4.8%). BrainSense survey use in clinic was also reported among *n* = 42 users: always in 22/42 (52.4%), most of the time in 15/42 (35.7%), occasionally in 4/42 (9.5%), and never in 1/42 (2.4%). Thus, BrainSense surveys were performed always or most of the time by 37/42 respondents (88.1%) (Supplementary Tables 27–29).

The purpose of conducting a BrainSense survey was captured as a multi-select item among respondents answering that question (*n* = 41). The most frequently selected purpose was to assist with contact selection/monopolar review (35/41, 85.4%), followed by determining which electrode pairs would be best to sense from (27/41, 65.9%) and determining whether frequencies of interest were present (18/41, 43.9%) (Table 2).

**Table 2:** Purpose of conducting BrainSense survey.

Variable	Number of respondents (n)	Percentage (%)
*What is the purpose of conducting a BrainSense Survey? (N = 41)*		
To assist with contact selection/monopolar review	35	85.4
To determine which pairs of electrodes will be best to sense from	27	65.9
To determine if any frequencies of interest are present	18	43.9

Purpose of conducting a BrainSense survey among respondents with available responses (*n* = 41). This was a multi-select item; percentages represent the proportion selecting each purpose and do not sum to 100%. The most frequently selected purpose was to assist with contact selection/monopolar review, followed by determining which electrode pairs would be best to sense from, and determining whether frequencies of interest were present.

BrainSense influence on programming adjustment was reported as a single-choice item among current users (*n* = 42): yes in 25/42 (59.5%), sometimes in 15/42 (35.7%), and no in 2/42 (4.8%). Features influencing programming adjustment were captured as a multi-select item among those with available responses (*n* = 40), with Survey most frequently selected (34/40, 85.0%), followed by Streaming (19/40, 47.5%), Events (13/40, 32.5%), and Timeline (11/40, 27.5%).

BrainSense influence on medication adjustment was reported among current users (single-choice, *n* = 42): yes in 17/42 (40.5%), sometimes in 18/42 (42.9%), and no in 7/42 (16.7%). Among respondents reporting BrainSense-informed medication adjustment features (multi-select, *n* = 35), the most frequently selected features were Events (17/35, 48.6%), Streaming (16/35, 45.7%), Timeline (14/35, 40.0%), and Survey (8/35, 22.9%) (Table 3).

**Table 3:** BrainSense influence on programming and medication adjustment.

Variable	Number of respondents (n)	Percentage (%)
*Does BrainSense influence programming adjustment? (N = 42)*		
Yes	25	59.5
Sometimes	15	35.7
No	2	4.8
*Which feature of BrainSense influences programming adjustment? (select one or more) (N = 40)*		
Survey	34	85
Streaming	19	47.5
Events	13	32.5
Timeline	11	27.5
*Does BrainSense influence medication adjustment? N = 42*		
Yes	17	40.4
Sometimes	18	42.9
No	7	16.6
*Which feature of BrainSense influences medication adjustment? (N = 35)*		
Events	17	48.5
Streaming	16	45.7
Timeline	14	40
Survey	8	22.8

Programming and medication adjustment with BrainSense. BrainSense-related programming and medication adjustment items were analysed using item-specific denominators restricted to current BrainSense users or to respondents with available feature-level responses, as applicable. Single-choice items use mutually exclusive categories. Multi-select feature items use the denominator of respondents with available feature-level responses and may total >100%.

Usefulness of the BrainSense threshold feature in identifying over- or under-treatment after initial programming was reported among current users (single-choice, *n* = 42): yes in 17/42 (40.5%), occasionally in 13/42 (31.0%), no in 9/42 (21.4%), and not aware of the threshold feature in 3/42 (7.1%) (Supplementary Table 30).

Adaptive DBS perception items were answered by all respondents (*n* = 50). Intention to incorporate adaptive DBS into clinical practice was reported as strongly agree by 28/50 (56.0%), agree by 17/50 (34.0%), and neutral by 5/50 (10.0%). Expected impact on outcomes was reported as definitely by 24/50 (48.0%), maybe by 22/50 (44.0%), and cannot comment by 4/50 (8.0%) (Supplementary Tables 31, 32).

### 3.7 Assessment of suboptimal response and lead re-evaluation

**Figure 6:**
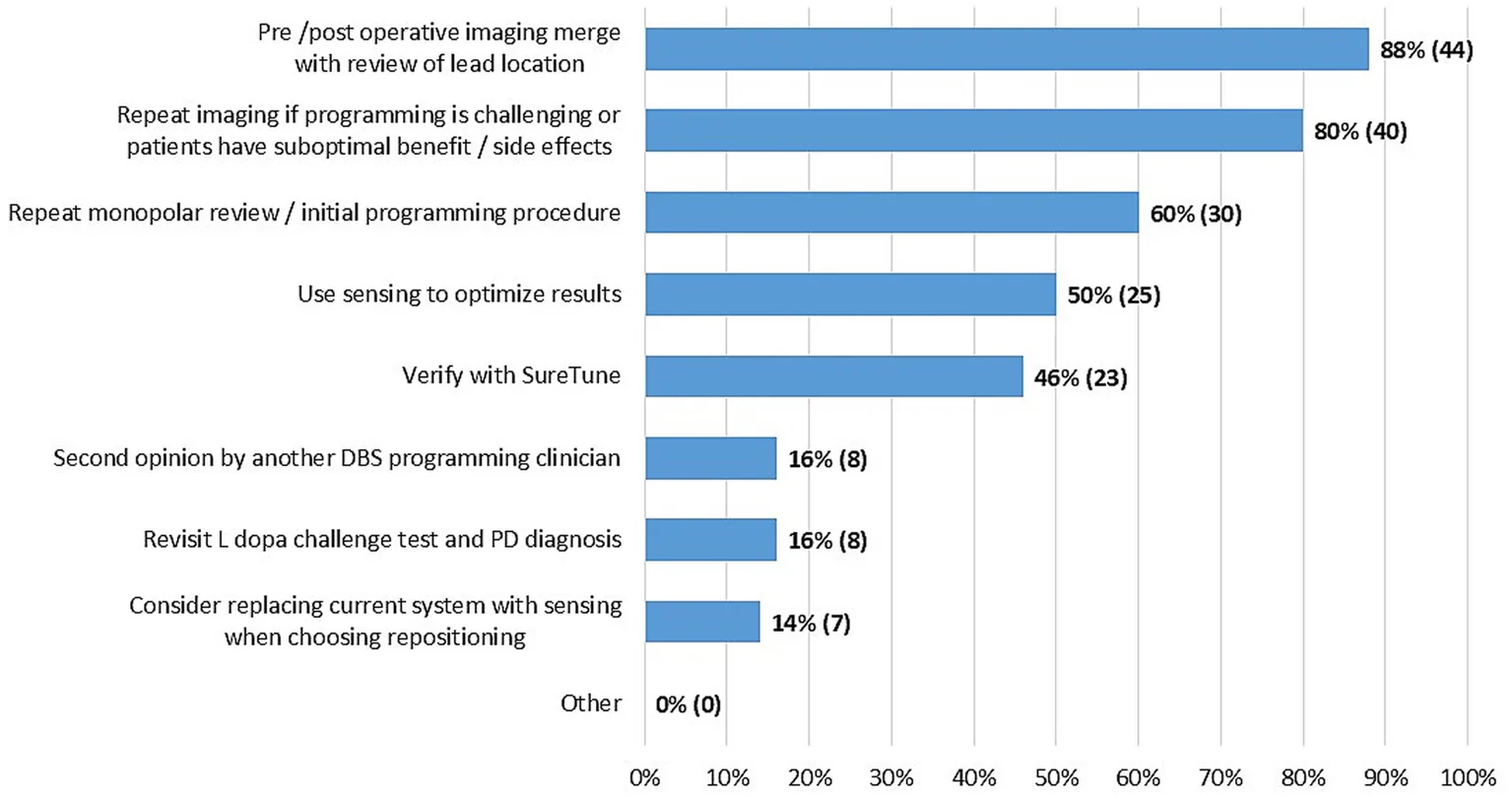
Strategies used to evaluate suboptimal response and determine whether lead re-evaluation may be required (*N* = 50). Approaches to suboptimal response were captured as a multi-select item and reported by all respondents (*n* = 50). Bars represent the proportion of respondents selecting each strategy. The most frequently selected approaches were pre−/post-operative imaging merge with review of lead location (44/50, 88.0%) and repeat imaging when programming was challenging or benefit remained suboptimal or side effects persisted (40/50, 80.0%). Percentages reflect option-level selections and do not sum to 100%.

Assessment of suboptimal response was reported as a multi-select item by all respondents (*n* = 50), and percentages therefore reflect the proportion selecting each strategy rather than mutually exclusive categories. The most frequently selected strategies were pre−/post-operative imaging merge with review of lead location (44/50, 88.0%) and repeat imaging when programming was challenging or therapeutic benefit remained suboptimal or side effects persisted (40/50, 80.0%). Repeat monopolar review was selected by 30/50 respondents (60.0%), sensing-based optimisation by 25/50 (50.0%), and SureTune verification by 23/50 (46.0%) (Figure 6; Supplementary Table 33). Ranking (on a scale of most likely to least likely) for the strategies to determine lead positioning has been presented in Supplementary Table 34.

### 3.8 Exploratory respondent-level workflow analysis

To complement item-level reporting, we performed an exploratory respondent-level pattern analysis using prespecified workflow domains rather than formal clustering. The domains were non-mutually exclusive and intended to identify broad workflow tendencies.

A clinically anchored conventional programming profile was identified in 13/50 respondents (26.0%), defined as use of traditional monopolar review without meeting criteria for a sensing-integrated workflow. A sensing-integrated programming profile was identified in 26/50 respondents (52.0%), defined by current BrainSense use, early initiation (at initial or first programming), BrainSense survey use always/most of the time, and BrainSense-influenced programming decisions (Table 4).

**Table 4:** Exploratory respondent-level workflow domains among DBS programmers in India (*N* = 50).

Exploratory workflow domain	Operational definition	Number of respondents (n)	Percentage (%)
Clinically anchored conventional programming	Traditional monopolar review used as part of initial programming, without meeting sensing-integrated criteria because BrainSense was absent/planned only, late/conditional, infrequent, or did not materially influence programming	13/50	26.0
Sensing-integrated programming	Current BrainSense use, early initiation (initial/first programming), BrainSense survey used always/most of the time, and BrainSense influences programming	26/50	52.0
Technology-assisted troubleshooting	Use of ≥3 advanced troubleshooting tools for suboptimal response: imaging merge, repeat imaging, sensing-based optimisation, SureTune verification	29/50	58.0
Structured follow-up-oriented workflow	Follow-up every 2 weeks or monthly until optimisation, off-medication programming always/almost always, and medication reduction guided by OFF-medication UPDRS ≥30% and/or BrainSense data	21/50	42.0

Domains were exploratory, prespecified, and non-mutually exclusive; therefore, totals exceed 100%. Formal clustering was not performed because of modest sample size and mixed survey item structure.

A technology-assisted troubleshooting profile was identified in 29/50 respondents (58.0%), defined by use of at least three advanced troubleshooting strategies for suboptimal response (pre−/post-operative imaging merge, repeat imaging, sensing-based optimisation, and SureTune verification). A structured follow-up-oriented workflow was identified in 21/50 respondents (42.0%), defined by planned follow-up every 2 weeks or monthly until optimisation, asking patients to come off dopaminergic medication always/almost always for subsequent programming visits, and use of at least one objective or technology-informed criterion for medication reduction (OFF-medication UPDRS improvement ≥30% and/or BrainSense data).

Overall, 31/50 respondents (62.0%) met at least two exploratory workflow domains and 13/50 (26.0%) met three domains, indicating substantial overlap across workflow styles rather than distinct, isolated practice archetypes. The most frequent overlap was between sensing-integrated programming and technology-assisted troubleshooting (20/50, 40.0%). An exploratory workflow/technology-integration score (range 0–9) had a mean of 6.4 ± 2.5 and median of 7 (IQR 6–8); 31/50 respondents (62.0%) fell into the high-score category (7–9), 11/50 (22.0%) into the intermediate category (4–6), and 8/50 (16.0%) into the low category (0–3). Exploratory respondent-level workflow domains among DBS programmers have been summarised in Table 3.

### 3.9 Exploratory subgroup analysis on association between advanced programming tools such as BrainSense and OptiStim

**Figure 7:**
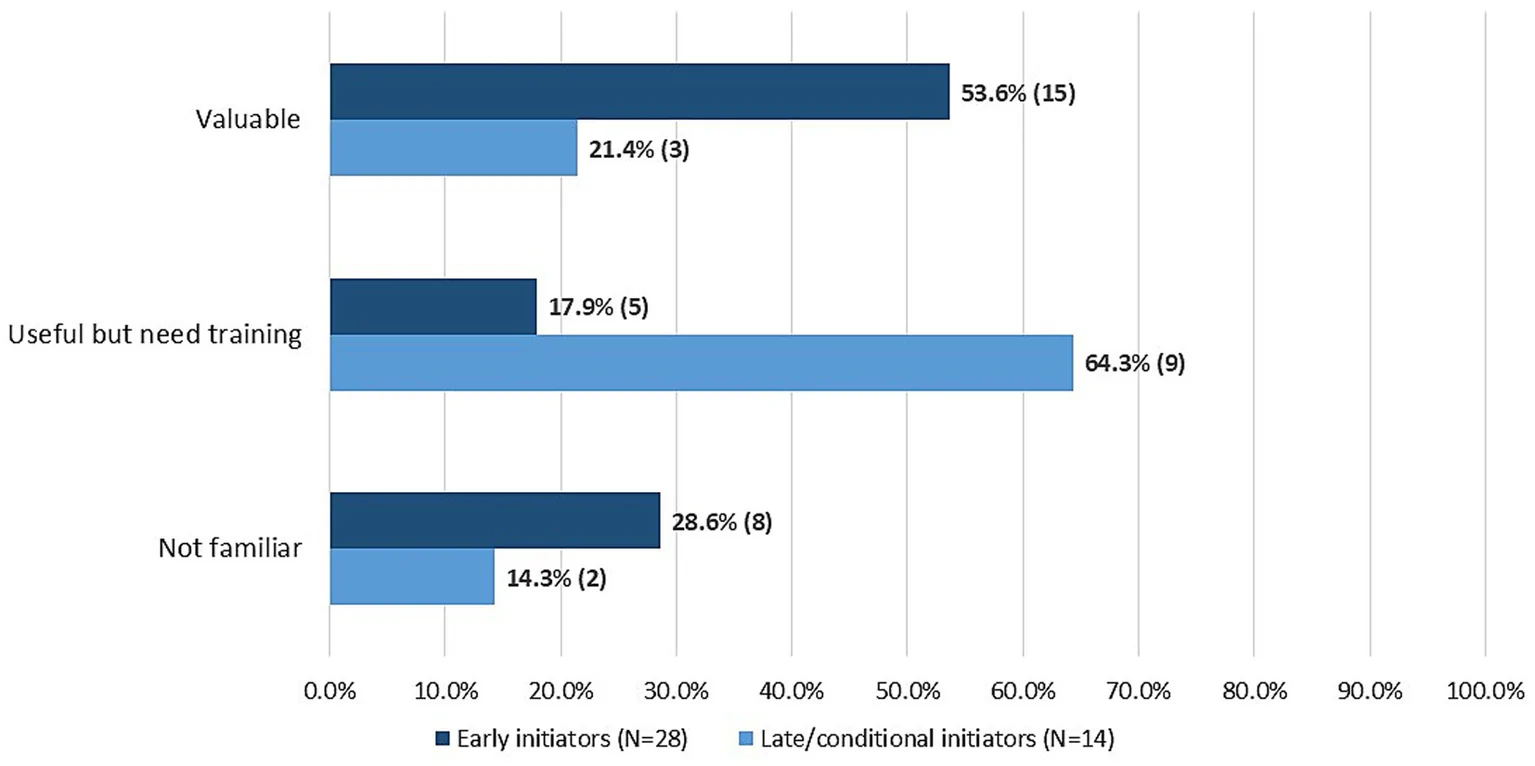
Association between timing of BrainSense initiation and perceived OptiStim maturity among early initiators and late/conditional initiators of DBS. Exploratory subgroup analysis among current BrainSense users with available initiation data compared early initiators, defined as initiation at initial or first programming (*n* = 28), with late/conditional initiators, defined as initiation at subsequent sessions or only when clinical programming results were suboptimal (*n* = 14). Among early initiators, OptiStim was reported as valuable by 15/28 (53.6%), useful but requiring training by 5/28 (17.9%), and not familiar by 8/28 (28.6%). Among late/conditional initiators, corresponding values were 3/14 (21.4%), 9/14 (64.3%), and 2/14 (14.3%), respectively. Percentages are calculated within subgroup.

Exploratory subgroup analysis examined the association between timing of BrainSense initiation and perceived OptiStim maturity among current BrainSense users with available initiation data. Early initiators, defined as respondents initiating BrainSense at initial or first programming, comprised 28 respondents, while late/conditional initiators, defined as initiation at subsequent sessions or only when clinical programming was suboptimal, comprised 14 respondents. Among early initiators (*n* = 28), OptiStim was reported as valuable by 15/28 (53.6%), useful but requiring training by 5/28 (17.9%), and not familiar by 8/28 (28.6%). Among late/conditional initiators (*n* = 14), corresponding values were 3/14 (21.4%), 9/14 (64.3%), and 2/14 (14.3%) (Figure 7).

## 4 Discussion

This multicentric clinician survey provides a descriptive snapshot of contemporary DBS programming practices for PD across India, spanning heterogeneous practice environments and varying stages of adoption of newer programming technologies. Importantly, the patterns described reflect self-reported routine practice and should not be interpreted as recommendations, evidence of superiority, or endorsement of majority responses as optimal standards of care.

Postoperative programming is an important modifiable factor that affects DBS outcomes and the long-term therapeutic response to DBS. Individualized programming strategies and standardized algorithms were also identified as key to maximizing the therapeutic benefit while keeping stimulation-induced adverse effects to a minimum (8, 9). While technology has improved in DBS hardware, the use of programming in DBS is still largely clinician-based, with significant variation in patient outcomes.

These have been overcome in recent years by some technological advances. Directional leads allow current steering and potentially enable a larger therapeutic window, providing a lower level of stimulation needed to achieve therapeutic response, allowing more targeted stimulation of the target structure while minimizing off-target effects. Krauss et al. (10) reported a growing use of and positive attitudes toward directional electrodes among surveyed European DBS experts, while acknowledging programming complexity and greater workload for clinicians as its challenges. These findings are consistent with the evidence summarized by Koeglsperger et al. (8), who reported that segmented (directional) DBS electrodes enable steering of the electric field toward the intended neural target while minimizing stimulation of adjacent structures associated with adverse effects. By restricting current spread and increasing the precision of stimulation delivery, directional leads may improve stimulation efficiency and broaden the therapeutic window, defined as the range between the threshold for clinical benefit and the threshold for stimulation-induced side effects. Furthermore, the authors suggested that directional stimulation may partially compensate for minor deviations in lead placement and potentially reduce energy requirements by achieving therapeutic effects at lower stimulation amplitudes.

Issues of access to DBS care have also come to the fore. Some of the factors that are found to limit the use of DBS in Asia and Oceania identified by Zhang et al. (11) were limited infrastructure, lack of adequate multidisciplinary expertise, and inadequate referrals. Similarly, Mahajan et al. (12) have also noted significant global variations in patient selection, target selection and postoperative management, reflecting the ongoing variability in DBS practices despite the presence of expert guidelines and international consensus statements.

Different ways to deal with these barriers are being explored, such as remote programming. Wan et al. (13) showed that remote programming was shown to be clinically equivalent to traditional (face-to-face) programming and decreased travel time and cost. The interest in telemedicine-based DBS management was also clearly demonstrated in the survey performed by Krauss et al. (10), which found that remote programming was one of the most promising technological advancements in the future.

### 4.1 Overall trends in Indian DBS programming practice

Across responses, DBS programming in India appears grounded in structured clinical reasoning, with newer technologies incorporated as additive layers rather than wholesale replacements of established workflows. Many clinicians described initial programming approaches that combine systematic clinical mapping, such as monopolar review, with sensing-based or verification tools. This suggests that explainability, reproducibility, and direct clinical observation remain central, even in technology-enabled settings. A second defining feature is heterogeneity across targets, activation timelines, follow-up cadence, medication adjustment practices, and depth of sensing integration, indicating that real-world DBS care in India consists of multiple locally adapted pathways rather than a single dominant model.

### 4.2 Practice environment and clinical context

Respondents represented a broad cross-section of DBS delivery settings, with most practicing in corporate hospitals, followed by government institutions, private medical colleges, and standalone hospitals. Monthly PD clinic volumes spanned a wide range, while most clinicians reported relatively low numbers of new DBS patients presenting for initial programming. This combination suggests that DBS programming workflows are implemented under diverse throughput pressures, with varying opportunities for repeated exposure to early post-implant programming. Such contextual factors are likely to influence clinician confidence, efficiency, and adoption of advanced programming and sensing capabilities. Notably, features such as directional current steering (OptiStim/Shapelock) and BrainSense tools (Survey, Streaming, Timeline, and Events) were used by a substantial proportion of respondents, suggesting that technology-enabled programming is increasingly being integrated into routine DBS practice.

### 4.3 Target selection and timing of initial programming

In our study, most respondents reported STN as their commonly used DBS target, whereas GPi was selected less frequently. However, this finding should not be interpreted as evidence of preferential practice quality or target superiority. Contemporary evidence supports individualized target selection based on patient characteristics rather than a universal preference for one nucleus. Selection between STN and GPi is guided by symptom profile, expected medication reduction, dyskinesia burden, cognitive and psychiatric status, age, and patient priorities. Consequently, the observed variability in target selection likely reflects appropriate patient-centred decision-making rather than inconsistency of care, with contemporary DBS practice increasingly emphasizing precision target selection over uniform target preference (14, 15).

The physiological roles of STN and GPi also influence subsequent programming strategies. Our study results show STN DBS is often favoured when reduction of dopaminergic medication burden is an important therapeutic goal and provides robust improvement in motor fluctuations and tremor. However, STN stimulation may require closer attention to stimulation-related cognitive, speech, and gait effects. In contrast, GPi DBS is frequently preferred in patients with troublesome dyskinesia or where preservation of cognitive and neuropsychiatric function is a greater priority. GPi stimulation offers strong antidyskinetic benefits with greater flexibility for medication adjustment and is often considered easier to program because medication reduction is not obligatory. These complementary characteristics underscore why programming strategies should remain individualized according to therapeutic goals rather than target alone (14).

Although the present survey did not capture target-specific programming workflows, medication adjustment practices demonstrated a tendency toward earlier reduction of dopaminergic therapy following STN DBS, whereas respondents reported greater flexibility after GPi DBS. These observations are consistent with the established therapeutic objectives associated with each target and should be interpreted descriptively rather than as comparative evidence of clinical effectiveness. Overall, the survey findings reinforce that differences in programming approaches are likely to reflect individualized therapeutic priorities rather than differences in the quality of clinical practice (14, 15).

### 4.4 Initial programming strategies and the role of advanced tools

Initial programming strategies remained strongly anchored in monopolar review-based reasoning. The most frequently reported approach combined monopolar review with sensing-enabled tools which was followed by monopolar review alone. This pattern aligns with a layered confidence model, in which clinical examination and systematic parameter exploration establish baseline settings, while sensing, imaging, or verification tools are used to refine decisions or reduce uncertainty rather than supplant foundational logic (15).

### 4.5 Adoption of directional stimulation and advanced features

Advanced programming features demonstrated a clear gradient of adoption rather than a binary use/non-use pattern. For tools such as OptiStim/ShapeLock, some clinicians described clear value in tailoring therapy, others acknowledged potential utility but highlighted the need for additional training, and a substantial minority reported limited familiarity. Hesitancy appeared driven more by implementation friction- time burden, cognitive load, training needs, and workflow disruption- than by rejection of the concept of current steering or field shaping. Collectively, these findings suggest that adoption of advanced features is influenced as much by clinic flow compatibility and confidence as by technological availability.

### 4.6 Symptom-specific optimisation strategies

Across core motor symptoms, clinicians most often reported beginning optimisation with simple, explainable levers. For tremor, frequency and amplitude adjustments predominated; for bradykinesia and rigidity, amplitude changes were most frequently selected, with other parameters used less consistently. These distributions suggest a pragmatic sequencing style, prioritising direct, easily interpretable adjustments before escalating complexity, rather than rigid adherence to symptom-specific algorithms.

The preference for frequency modulation observed in our survey is consistent with current understanding of DBS physiology and contemporary programming practice. High-frequency stimulation (typically >100 Hz) remains the standard approach for controlling tremor by suppressing pathological oscillatory activity within the basal ganglia–thalamocortical network and disrupting abnormal neuronal synchronisation. Consequently, clinicians commonly modify stimulation frequency when tremor persists despite adequate amplitude adjustment, particularly when attempting to optimize tremor control while minimizing stimulation-related adverse effects (8, 15).

Intralingual, high stimulation frequencies are known to be effective in suppressing tremor and are widely used to treat refractory tremor. In contrast, low stimulation frequencies are being investigated in some patients with axial symptoms and freezing of gait, possibly through differential modulation of locomotor and brainstem networks. Overall, the results indicate that frequency adjustments are preferred when the physiologic nature of the symptom is particularly frequency dependent. In contrast, greater spatial and temporal precision is required when the therapeutic window is narrower, and programming is simpler.

In contrast, freezing of gait and other axial symptoms represent more complex therapeutic challenges. Increasing evidence suggests that selected patients may benefit from lower-frequency stimulation (approximately 60–80 Hz) or variable-frequency stimulation, which may differentially modulate locomotor and brainstem networks compared with conventional high-frequency stimulation. Clinical studies have demonstrated improvements in gait performance and freezing episodes in selected patients receiving low- or variable-frequency stimulation, although responses remain heterogeneous and individualized programming remains essential (16, 17).

Therefore, the predominance of frequency-based programming for tremor and freezing of gait observed in our survey likely reflects an evidence-informed, symptom-specific programming approach. In contrast, amplitude adjustment remained the preferred strategy for bradykinesia and rigidity, consistent with established DBS programming principles that recommend amplitude as the first stimulation parameter to optimize because it has the greatest influence on the volume of tissue activated and overall motor benefit (15, 16).

### 4.7 Managing side effects and complex motor phenomena

In situations where the primary issue was the side effects of stimulation or narrow therapeutic windows, there was a change in response patterns to strategies that would change the context in which stimulation was prescribed and/or implemented. Side effects with dyskinesia, speech, and swallowing were often addressed with amplitude modulation, contact changes, bipolar configurations, pulse width modulation, and directionality, with combinations used. Freezing of gait showed a unique profile, with significant frequency adjustments, interleaving contacts, and contact-level modifications, indicating a preference for exploring more complex configurations for symptoms that are not always easily stabilised.

### 4.8 Medication adjustment practices

Medication adjustment following DBS programming emerged as predominantly clinician- and patient-judgment driven, supported variably by objective anchors. Patient-perceived improvement, OFF-medication UPDRS reduction thresholds, side effects, and visual assessment without formal scales were commonly cited, while sensing-derived inputs were used by a smaller proportion. Timelines for medication reduction varied and differed by target, with greater flexibility reported after GPi programming. These findings should be interpreted as mapping real-world decision heuristics rather than validating any specific taper strategy, as they are likely shaped by follow-up access, patient travel, clinician risk tolerance, and local monitoring capacity.

### 4.9 Role of sensing technologies in clinical decision-making

Sensing-enabled DBS was widely represented, with most respondents reporting current or planned routine BrainSense use. However, both timing of initiation and feature utilisation varied substantially. Notably, different BrainSense features appeared to serve distinct cognitive roles. Survey-derived outputs were most frequently used to inform programming decisions, whereas longitudinal features such as Events, Streaming, and Timeline were more commonly cited as influencing medication adjustments. This separation aligns with how clinicians integrate physiology-derived data into existing workflows, reserving longitudinal context for state-dependent or slower clinical decisions. The recent introduction of electrode identifier capabilities in India, enabling monopolar recordings using distal references, may further influence workflow efficiency and contact selection approaches, although real-world impact remains to be seen (15).

### 4.10 Approaches to suboptimal response and lead re-evaluation

When faced with suboptimal outcomes, clinicians most often described a verification-heavy pathway before considering invasive escalation. Imaging-based lead localisation and repeat imaging in challenging cases were frequently selected, alongside repeat monopolar review and sensing-based optimisation. Navigation and verification tools were commonly used as de-risking utilities to reduce uncertainty and exhaust programmable options before contemplating lead revision.

### 4.11 Exploratory respondent-level workflow analysis

The respondent-level exploratory analysis adds interpretive context to the option-level descriptive findings by suggesting that DBS programming variability in India may be better conceptualised as overlapping workflow styles rather than isolated item-level decisions. In particular, sensing-integrated programming and technology-assisted troubleshooting frequently co-occurred, while a substantial proportion of respondents also demonstrated structured follow-up workflows. These findings support the interpretation that practice heterogeneity reflects graded technology-adoption and workflow-integration states rather than discrete, mutually exclusive programming archetypes. Because these exploratory domains were derived from a modest sample and were not generated through formal clustering, they should be viewed as hypothesis-generating rather than as validated phenotypes. Future larger studies with prospectively designed respondent-level analytic frameworks should evaluate whether such workflow combinations are associated with programming efficiency, therapeutic stability, or patient outcomes.

### 4.12 Exploratory subgroup analysis on association between advanced programming tools such as BrainSense and OptiStim

The exploratory subgroup analysis further supports the interpretation that adoption of advanced DBS programming tools occurs along a maturity gradient rather than as a binary use/non-use pattern. Respondents who initiated BrainSense earlier in the programming pathway more frequently reported OptiStim/ShapeLock as valuable for therapy tailoring, whereas late or conditional BrainSense initiators more often described the feature as useful but requiring additional training. This pattern may reflect differences in cumulative exposure, confidence with sensing-enabled workflows, and integration of advanced tools into routine clinic processes, rather than a causal relationship between BrainSense initiation timing and perceived OptiStim utility. Importantly, a proportion of early initiators also reported lack of familiarity with OptiStim/ShapeLock, indicating that adoption of one advanced feature does not necessarily imply uniform familiarity with all available programming capabilities. These findings reinforce the broader observation that technology-enabled DBS programming in India is heterogeneous and likely shaped by training exposure, workflow compatibility, and clinician confidence, highlighting the need for structured, practical education around the integration of sensing, current-shaping, and verification tools into routine programming pathways.

### 4.13 Readiness for and perceptions of adaptive (closed-loop) DBS in India

The strong interest in adaptive DBS observed in our survey reflects a clinically receptive environment for the next phase of DBS technology adoption in India. Most respondents indicated willingness to incorporate adaptive DBS into practice and anticipated potential patient benefit, suggesting that Indian DBS programmers are already thinking beyond conventional continuous stimulation toward more personalised, physiology-informed therapy. Recent ADAPT-PD publications provide timely external context for these expectations: the methodology report showed that local field potential signals suitable for adaptive algorithms could be identified in a high proportion of enrolled patients, and the subsequent clinical report demonstrated that long-term at-home adaptive DBS was generally tolerable, safe, and able to maintain comparable on-time without troublesome dyskinesia relative to stable continuous DBS in selected patients (5, 18).

These findings support the biological and clinical plausibility of adaptive stimulation and help explain the optimism expressed by clinicians in our survey. In the Indian context, the pace and consistency of adoption will likely depend on practical enablers such as structured training, patient selection frameworks, programming time, follow-up capacity, and clinician confidence in interpreting sensing-derived data. Thus, our findings should be viewed as documenting early clinician readiness for adaptive DBS, while highlighting the need for implementation-focused education and local real-world experience to support its integration into routine DBS programming workflows.

### 4.14 Practice variability, training gaps, and opportunities for standardization

Across multiple domains, the survey highlights practice variability that is better understood as reflecting different stages of technology adoption rather than differences in care quality. For example, responses on OptiStim indicate that a majority of clinicians fall into “needs training” or “not familiar” categories, while use of BrainSense thresholding ranged from regular to occasional or non-use. Together, these patterns suggest that the diffusion of advanced DBS tools may be progressing faster than the development of structured, practical onboarding around when, why, and how to integrate higher-complexity features into time-constrained clinics.

### 4.15 Implications for clinical practice, training, and future DBS Care in India

A subgroup perspective further illustrates how confidence and workflow integration shape adoption. Clinicians who initiated BrainSense earlier in the programming pathway more frequently described OptiStim as valuable, whereas late or conditional initiators more often viewed it as useful but requiring additional training. This apparent maturity gradient likely reflects differences in exposure, familiarity, and clinic structure rather than causal relationships between technologies, reinforcing the importance of training and workflow alignment.

### 4.16 Study limitations and areas for future research

This study has several limitations that should be considered when interpreting the findings. First, the survey employed purposive sampling with voluntary participation, which makes it susceptible to selection bias. Although respondents were drawn from 37 DBS centres across 18 cities and all major Indian regions were represented, the sample was purposive and regionally imbalanced, with a predominance of respondents from South India. Therefore, the findings should be interpreted as a geographically distributed multicentre clinician survey rather than as nationally representative estimates of DBS programming practice in India.

Clinicians from technologically advanced, academically affiliated, or high-volume DBS centres may have been more likely to participate, potentially resulting in an overrepresentation of centres with greater access to advanced programming technologies. Nevertheless, the inclusion of respondents from all four geographical regions of India, diverse practice settings, and centres with varying patient volumes suggests that the survey captured a broad spectrum of contemporary DBS programming practices.

Second, the survey was designed to characterise clinician-reported programming strategies and perceptions and did not collect patient-level clinical outcomes. Consequently, the findings cannot be linked to treatment efficacy, safety, quality of life, or other patient-centred outcomes, and no conclusions can be drawn regarding the comparative effectiveness of individual programming approaches. The questionnaire was designed to characterize overall programming practices and did not capture programming workflows separately according to DBS target (STN versus GPi), limiting the ability to perform target-specific comparative analyses.

Third, although information on the use of advanced programming technologies was collected, responses were not stratified by specific DBS devices or manufacturers. Differences in device capabilities, proprietary programming algorithms, sensing technologies, and software platforms may influence programming workflows and adoption patterns and could therefore have contributed to the observed heterogeneity in practice.

Finally, multiple respondents originated from some institutions, introducing the possibility of centre-level clustering and non-independence of responses. Although most participating centres (30/37, 81.1%) contributed a single respondent, five centres (13.5%) contributed two respondents, and two centres (5.4%) contributed three respondents. Consequently, institutional preferences and local programming protocols may have influenced some of the observed findings. Furthermore, as the survey relied on self-reported practices, the findings may also be subject to recall and reporting biases.

Therefore, the results should be interpreted as reflecting the practices and perceptions of the participating cohort and should not be considered a fully representative assessment of all DBS programming practices in India.

## 5 Conclusion

In this a geographically distributed multicentre survey of DBS programmers across India, we provide a descriptive snapshot of contemporary real-world programming practices for Parkinson’s disease, spanning initial activation workflows, symptom-specific optimisation strategies, medication adjustment approaches, use of sensing-enabled technologies, and approaches to suboptimal response. The findings highlight substantial practice variability across multiple domains, while also revealing shared underlying clinical workflows- most notably, the continued centrality of structured, clinically driven programming approaches, with advanced tools commonly integrated as adjuncts rather than replacements. These results are intended to document current practice patterns and adoption states and should not be interpreted as recommendations or as evidence that more frequently reported practices are superior or guideline-concordant.

We also observed broad uptake of sensing-enabled systems and strong clinician interest in adaptive DBS, reflecting a rapidly evolving technology landscape alongside emerging external evidence supporting the feasibility of long-term adaptive stimulation in selected patients. Together, these data underscore opportunities for outcome-linked real-world research in Indian care settings and for context-appropriate training initiatives that support safe, consistent integration of newer programming features within routine clinical workflows.

## Data Availability

The original contributions presented in the study are included in the article/Supplementary material, further inquiries can be directed to the corresponding author/s.
