# Supplementary material for: Real-world deep brain stimulation programming practices for Parkinson’s disease in India: variability, technology adoption, and emerging trends from a multicentre clinician survey across India
Source: Front Neurol. 2026 Aug 31;17:1892612. doi: 10.3389/fneur.2026.1892612 (PMC13572247; doi:10.3389/fneur.2026.1892612)
Supplement: Supplementary file 1 [file Data_Sheet_1.PDF]

Real World Practices in DBS Programming for Parkinson's Disease

To capture expert insights on DBS Programming Practices for treatment of Parkinson's Disease

* Required

1. Name *

2. Which city do you have your practice in? *

3. Specialization *

4. Years of experience in DBS for Parkinson's disease *

5. Please describe your practice as one of the following. *

- ◯ Government hospital
- ◯ Corporate hospital
- ◯ Non corporate/ Standalone hospital
- ◯ Private medical college

6. How many Parkinson's Disease patients in any given month do you see? *

- ◯ 1-15
- ◯ 16-30
- ◯ 31-50
- ◯ >50

7. How many new PD patients in any given month do you see for initial DBS programming? *

- ◯ 1-5
- ◯ 6-10
- ◯ 11-15
- ◯ >15

8. Which nucleus is commonly used for lead placement in DBS for PD, beyond consideration of patient factors? *

- ◯ STN
- ◯ Gpi
- ◯ Other

9. How long do you wait after IPG implantation to begin the stimulation through initial programming session? *

- ◯ 1-2 weeks
- ◯ 3-4 weeks
- ◯ 1-3 months
- ◯ >3 months
- ◯ Other

10. In your setup, who performs the initial programming? *

- ☐ Neurologist- Movement disorder fellows
- ☐ Neurologist- Movement disorder consultants
- ☐ Neurosurgeon
- ☐ PD Nurse

11. How much time do you allocate for programming a patient with bilateral leads? *

- 30-60 minutes
- 61-90 minutes
- 91-120 minutes
- >120 minutes

12. Do you prefer ring stimulation or segmental stimulation to begin with? *

- Usually a ring electrode
- Usually a segmented electrode
- Ring or segmented electrode based on patient presentation

Initial programming practices

13. Which strategies do you use for initial programming of DBS? *

- [ ] Traditional monopolar review
- [ ] Physiological guided contact selection tools such as BrainSense
- [ ] A combination of monopolar review and BrainSense
- [ ] SureTune verification
- [ ] Other

14. Please rank the strategy for initial programming that you commonly use *

	Most frequent	Frequent	Least frequent	Not available
Traditional monopolar review	◯	◯	◯	◯
Physiological guided contact selection tools such as BrainSense	◯	◯	◯	◯
A combination of monopolar review and BrainSense	◯	◯	◯	◯
SureTune verification	◯	◯	◯	◯
Image guided	◯	◯	◯	◯

15. Have you utilized OptiStim and ShapeLock which allows you to assign unique amplitude values per electrode and modify configurations without resetting all amplitudes? *

◯ Yes, it is valuable in tailoring therapy

◯ It has utility but need training to implement this feature effectively

◯ Not familiar with this feature

◯ It does not have any utility for improving patient outcome

16. How does this feature optimize patient outcomes *

17. Why do you believe it does not have any utility? *

Optimization strategy for symptoms of PD

What is the optimization strategy you use for following symptoms

18. Dyskinesia- select upto 3 top optimization strategies *

Please select at most 3 options.

- [ ] Modify amplitude
- [ ] Modify frequency
- [ ] Modify pulse width
- [ ] Activate a segment
- [ ] Alter electrode location along the lead
- [ ] Switch to bipolar mode
- [ ] Change to interleaved stimulation
- [ ] Use OptiStim
- [ ] Switch to double monopolar mode

19. Tremor- select upto 3 top optimization strategies *

Please select at most 3 options.

- [ ] Modify amplitude
- [ ] Modify frequency
- [ ] Modify pulse width
- [ ] Activate a segment
- [ ] Alter electrode location along the lead
- [ ] Switch to bipolar mode
- [ ] Change to interleaved stimulation
- [ ] Use OptiStim
- [ ] Switch to double monopolar mode

20. Bradykinesia- select upto 3 top optimization strategies *

Please select at most 3 options.

- ☐ Modify amplitude
- ☐ Modify frequency
- ☐ Modify pulse width
- ☐ Activate a segment
- ☐ Alter electrode location along the lead
- ☐ Switch to bipolar mode
- ☐ Change to interleaved stimulation
- ☐ Use OptiStim
- ☐ Switch to double monopolar mode

21. Rigidity- select upto 3 top optimization strategies *

Please select at most 3 options.

- ☐ Modify amplitude
- ☐ Modify frequency
- ☐ Modify pulse width
- ☐ Activate a segment
- ☐ Alter electrode location along the lead
- ☐ Switch to bipolar mode
- ☐ Change to interleaved stimulation
- ☐ Use OptiStim
- ☐ Switch to double monopolar mode

22. Speech related side effects- select upto 3 top optimization strategies *

Please select at most 3 options.

- [ ] Modify amplitude
- [ ] Modify frequency
- [ ] Modify pulse width
- [ ] Activate a segment
- [ ] Alter electrode location along the lead
- [ ] Switch to bipolar mode
- [ ] Change to interleaved stimulation
- [ ] Use OptiStim
- [ ] Switch to double monopolar mode

23. Non-motor adverse effects- select upto 3 top optimization strategies *

Please select at most 3 options.

- [ ] Modify amplitude
- [ ] Modify frequency
- [ ] Modify pulse width
- [ ] Activate a segment
- [ ] Alter electrode location along the lead
- [ ] Switch to bipolar mode
- [ ] Change to interleaved stimulation
- [ ] Use OptiStim
- [ ] Switch to double monopolar mode

24. Swallowing related side effects- select upto 3 top optimization strategies *

Please select at most 3 options.

- [ ] Modify amplitude
- [ ] Modify frequency
- [ ] Modify pulse width
- [ ] Activate a segment
- [ ] Alter electrode location along the lead
- [ ] Switch to bipolar mode
- [ ] Change to interleaved stimulation
- [ ] Use OptiStim
- [ ] Switch to double monopolar mode

25. Freezing of gait- select upto 3 top optimization strategies *

Please select at most 3 options.

- [ ] Modify amplitude
- [ ] Modify frequency
- [ ] Modify pulse width
- [ ] Activate a segment
- [ ] Alter electrode location along the lead
- [ ] Switch to bipolar mode
- [ ] Change to interleaved stimulation
- [ ] Use OptiStim
- [ ] Switch to double monopolar mode

26. What are patients taught at initial programming? (select one or more) *

- [ ] How to turn off the device for procedures
- [ ] How to check battery
- [ ] How to charge rechargeable devices
- [ ] How to adjust individual parameters
- [ ] How to put device into MRI mode
- [ ] How to mark events on Percept device
- [ ] How to change between groups

Subsequent programming sessions

27. Following initial programming of DBS **STN**, when is the medication reduced? *

- ◯ At the time of the initial programming
- ◯ One week after initial programming
- ◯ One month after initial programming
- ◯ Three months after initial programming
- ◯ No set time for medication reduction

28. Following initial programming of DBS ***GPi,*** when is the medication reduced? *

- ◯ At the time of the initial programming
- ◯ One week after initial programming
- ◯ One month after initial programming
- ◯ Three months after initial programming
- ◯ No set time for medication reduction

29. What do you use to base your decision on medication reduction? (select one or more) *

- ☐ Patient perception of improvement
- ☐ OFF medication UPDRS score reduction by 30% or more
- ☐ Assessment of BrainSense data
- ☐ Patient reported side effects
- ☐ Patient report of skipping doses
- ☐ Visual assessment of patient response showing improvement without a formal scale
- ☐ Other

30. Rank the factor you use to base your decision on medication reduction *

	Most important	Important	Least important
Patient perception of improvement	◯	◯	◯
OFF medication UPDRS score reduction by 30% or more	◯	◯	◯
Assessment of BrainSense data	◯	◯	◯
Patient reported side effects	◯	◯	◯
Patient report of skipping doses	◯	◯	◯
Visual assessment of patient response demonstrating improvement without a formal scale	◯	◯	◯

31. Do you ask patients to come off dopaminergic medication for subsequent programming sessions? *

- ◯ Always
- ◯ Almost always
- ◯ Sometimes
- ◯ Never

32. What do you recommend for planning patient visits following initial programming? *

- ◯ Every 2 weeks post initial programming until stimulation is optimized
- ◯ Every month post initial programming until stimulation is optimized
- ◯ Every 2 months post initial programming until stimulation is optimized
- ◯ Every 3 months post initial programming until stimulation is optimized

BrainSense in programming

33. Are you currently using a BrainSense sensing-enabled DBS system? *

- ◯ Yes
- ◯ No
- ◯ Plan to use

34. When do you initiate BrainSense? *

- ◯ At initial programming
- ◯ After the initial programming- at second or subsequent programming sessions
- ◯ When results of clinical programming are not optimal

35. Do you perform a BrainSense survey in clinic? *

- ◯ Yes, always
- ◯ Most of the time
- ◯ Occasionally
- ◯ No, never

36. What is the purpose of conducting a BrainSense Survey? (Select one or more) *

- ☐ To assist with contact selection/ monopolar review
- ☐ To determine if any frequencies of interest are present
- ☐ To determine which pairs of electrodes will be best to sense from

37. Does BrainSense influence programming adjustment? *

- ◯ Yes
- ◯ Sometimes
- ◯ No

38. Which feature of BrainSense influences programming adjustment? (select one or more) *

- ☐ Survey
- ☐ Streaming
- ☐ Timeline
- ☐ Events

39. Rank the BrainSense features which influence programming adjustment? (Rank most to least) *

- Survey
- Streaming
- Timeline
- Events

40. Does BrainSense influence medication adjustment? *

- ◯ Yes
- ◯ Sometimes
- ◯ No

41. Which feature of BrainSense influences medication adjustment? (select one or more) *

- ☐ Survey
- ☐ Streaming
- ☐ Timeline
- ☐ Events

42. Has BrainSense **threshold feature** aided in identifying whether a patient is over treated or undertreated after initial programming? *

- ◯ Yes
- ◯ Occasionally
- ◯ No
- ◯ Not aware of threshold feature

Optimization of suboptimal patient response to DBS

Assuming expectations of patient are appropriate and target motor symptoms known to be helped by DBS are not being adequately treated....

43. For suboptimal patient response to DBS, what strategies are used to determine if lead repositioning is required? (select one or more) *

- ☐ Repeat monopolar review/ initial programming procedure
- ☐ Second opinion by another DBS programming clinician
- ☐ Pre-/post-operative imaging merge with review of lead location
- ☐ Repeat imaging if programming is challenging and patients are not achieving therapeutic benefit or are having side effects
- ☐ Use sensing to optimize results
- ☐ Verify with SureTune
- ☐ Consider replacing current system with sensing if choosing re-positioning:
- ☐ Revisit L-dopa challenge test and diagnosis of PD
- ☐ Other

44. For suboptimal patient response to DBS, rank the strategies you use to determine if lead repositioning is required? *

	Most likely	Likely	Least likely
Repeat monopolar review/ initial programming procedure	◯	◯	◯
Second opinion by another DBS programming clinician	◯	◯	◯
Pre-/post-operative imaging merge with review of lead location	◯	◯	◯
Repeat imaging if programming is challenging and patients are not achieving therapeutic benefit or are having side effects	◯	◯	◯
Use sensing to optimize results	◯	◯	◯
Verify with SureTune	◯	◯	◯
Consider replacing current system with sensing if choosing re-positioning	◯	◯	◯
Revisit L-dopa challenge test and diagnosis of PD	◯	◯	◯

Adaptive DBS

Closed loop stimulation for DBS

45. After the launch of Adaptive Deep Brain Stimulation in India, you intend to incorporate it into your clinical practice for treating PD patients *

- Strongly agree
- Agree
- Neutral
- Disagree
- Strongly disagree

46. Do you foresee Adaptive DBS leading to improved patient outcomes in PD management? *

- Definitely
- Maybe
- Unlikely
- Cannot comment at this point

This content is neither created nor endorsed by Microsoft. The data you submit will be sent to the form owner.

Microsoft Forms
