# Supplementary material for: Real-world deep brain stimulation programming practices for Parkinson’s disease in India: variability, technology adoption, and emerging trends from a multicentre clinician survey across India
Source: Front Neurol. 2026 Aug 31;17:1892612. doi: 10.3389/fneur.2026.1892612 (PMC13572247; doi:10.3389/fneur.2026.1892612)
Supplement: Supplementary file 2 [file Table_1.DOCX]

**SUPPLEMENT 2: Analysis of results**

**Q 1, 2, 3, 4 HCP name, city, specialty, years of experience**

Presented in the manuscript

**Table 1: Please describe your practice as one of the following. N=50**

|  | **Number of respondents (n)** | **Percentage (%)** |
| --- | --- | --- |
| **Govt** | 8 | 16 |
| **Corporate** | 32 | 64 |
| **Non corporate/standalone** | 4 | 8 |
| **Private medical college** | 6 | 12 |

**Table 2: How many Parkinson's Disease patients in any given month do you see? N=50**

|  | **Number of respondents (n)** | **Percentage (%)** |
| --- | --- | --- |
| **1-15** | 14 | 28 |
| **16-30** | 9 | 18 |
| **31-50** | 14 | 28 |
| **>5-** | 13 | 26 |

**Table 3: How many new PD patients in any given month do you see for initial DBS programming? N=50**

|  | **Number of respondents (n)** | **Percentage (%)** |
| --- | --- | --- |
| **1-5** | 39 | 78 |
| **6-10** | 8 | 16 |
| **11-15** | 2 | 4 |
| **>15** | 1 | 2 |

**Table 4: Which nucleus is commonly used for lead placement in DBS for PD, beyond consideration of patient factors? N=50**

|  | **Number of respondents (n)** | **Percentage (%)** |
| --- | --- | --- |
| **STN** | 42 | 84 |
| **Gpi** | 7 | 14 |
| **Other** | 1 | 2 |

**Table 5: How long do you wait after IPG implantation to begin the stimulation through initial programming sessions? N=50**

|  | **Number of respondents (n)** | **Percentage (%)** |
| --- | --- | --- |
| **1-2 weeks** | 25 | 50 |
| **3-4 weeks** | 14 | 28 |
| **1-3 months** | 3 | 6 |
| **>3 months** | 1 | 2 |
| **Other** | 7 | 14 |

**Table 6: In your setup, who performs the initial programming? N= 50**

|  | **Number of respondents (n)** | **Percentage (%)** |
| --- | --- | --- |
| **Neurologist- Movement disorder fellows** | 9 | 18 |
| **Neurologist- Movement disorder consultants** | 39 | 78 |
| **Neurosurgeon** | 9 | 18 |
| **PD nurse** | 0 | 0 |

**Table 7: How much time do you allocate for programming a patient with bilateral leads? N=50**

|  | **Number of respondents (n)** | **Percentage (%)** |
| --- | --- | --- |
| **30-60 minutes** | 21 | 42 |
| **61- 90 minutes** | 19 | 38 |
| **91-120 minutes** | 6 | 12 |
| **> 120 minutes** | 4 | 8 |

**Table 8: Do you prefer ring stimulation or segmental stimulation to begin with? N=50**

|  | **Number of respondents (n)** | **Percentage (%)** |
| --- | --- | --- |
| **Usually a ring electrode** | 30 | 60 |
| **Usually a segmented electrode** | 6 | 12 |
| **Ring or segmented electrode based on patient presentation** | 14 | 28 |

**Table 9: Which strategies do you use for initial programming of DBS? N=50**

|  | **Number of respondents (n)** | **Percentage (%)** |
| --- | --- | --- |
| **Traditional monopolar review** | 22 | 44 |
| **Physiological guided contact selection tools such as BrainSense** | 8 | 16 |
| **A combination of monopolar review and BrainSense** | 35 | 70 |
| **SureTune verification** | 9 | 18 |
| **Other** | 2 | 4 |

**Table 10: Please rank the strategy for initial programming that you commonly use. N=50**

|  | **Most frequent %** | **Frequent %** | **Least frequent %** | **Not available %** |
| --- | --- | --- | --- | --- |
| **Traditional monopolar** | **56** | **32** | **12** |  |
| **Physiological guided contact selection tools such as BrainSense** | **6** | **56** | **38** |  |
| **A combination of monopolar review and BrainSense** | **34** | **44** | **22** |  |
| **SureTune verification** | **10** | **24** | **44** | **22** |
| **Image guided** | **20** | **33.3** | **40** | **6.7** |

**Table 11: Have you utilized OptiStim and ShapeLock which allows you to assign unique amplitude values per electrode and modify configurations without resetting all amplitudes? N=50**

|  | **Number of respondents (n)** | **Percentage (%)** |
| --- | --- | --- |
| **Yes, it is valuable in tailoring therapy** | 19 | 38 |
| **It has utility but need training to implement this feature effectively** | 17 | 34 |
| **Not familiar with this feature** | 14 | 28 |
| **It does not have any utility for improving patient outcome** | 0 | 0 |

**Q 16 How does this feature optimize patient outcomes? N=36**

**Q 17 Why do you believe it does not have any utility? N=36**

**Table 12: Dyskinesia- select upto 3 top optimization strategies N=50**

|  | **Number of respondents (n)** |  | **Percentage (%)** |
| --- | --- | --- | --- |
| **Modify amplitude** | 47 |  | 94 |
| **Modify frequency** | 19 |  | 38 |
| **Modify pulse width** | 12 |  | 24 |
| **Activate a segment** | 9 |  | 18 |
| **Alter electrode location along the lead** | 18 |  | 36 |
| **Switch to bipolar mode** | 15 |  | 30 |
| **Change to interleaved stimulation** | 9 |  | 18 |
| **Use Optistim** | 13 |  | 26 |
| **Switch to double monopolar mode** | 2 |  | 4 |

**Table 13: Tremor- select upto 3 top optimization strategies N=50**

|  | **Number of respondents (n)** | **Percentage (%)** |
| --- | --- | --- |
| **Modify amplitude** | 39 | 78 |
| **Modify frequency** | 47 | 94 |
| **Modify pulse width** | 5 | 10 |
| **Activate a segment** | 10 | 20 |
| **Alter electrode location along the lead** | 19 | 38 |
| **Switch to bipolar mode** | 0 | 0 |
| **Change to interleaved stimulation** | 9 | 18 |
| **Use Optistim** | 4 | 8 |
| **Switch to double monopolar mode** | 11 | 22 |

**Table 14: Bradykinesia- select upto 3 top optimization strategies N=50**

|  | **Number of respondents (n)** | **Percentage (%)** |
| --- | --- | --- |
| **Modify amplitude** | 42 | 84 |
| **Modify frequency** | 19 | 38 |
| **Modify pulse width** | 19 | 38 |
| **Activate a segment** | 13 | 26 |
| **Alter electrode location along the lead** | 19 | 38 |
| **Switch to bipolar mode** | 8 | 16 |
| **Change to interleaved stimulation** | 7 | 14 |
| **Use Optistim** | 7 | 14 |
| **Switch to double monopolar mode** | 10 | 20 |

**Table 15: Rigidity- select upto 3 top optimization strategies N=50**

|  | **Number of respondents (n)** | **Percentage (%)** |
| --- | --- | --- |
| **Modify amplitude** | 45 | 90 |
| **Modify frequency** | 20 | 40 |
| **Modify pulse width** | 21 | 42 |
| **Activate a segment** | 14 | 28 |
| **Alter electrode location along the lead** | 18 | 36 |
| **Switch to bipolar mode** | 3 | 6 |
| **Change to interleaved stimulation** | 6 | 12 |
| **Use Optistim** | 7 | 14 |
| **Switch to double monopolar mode** | 8 | 16 |

**Table 16: Speech related side effects- select upto 3 top optimization strategies N=50**

|  | **Number of respondents (n)** | **Percentage (%)** |
| --- | --- | --- |
| **Modify amplitude** | 33 | 66 |
| **Modify frequency** | 30 | 60 |
| **Modify pulse width** | 17 | 34 |
| **Activate a segment** | 12 | 24 |
| **Alter electrode location along the lead** | 18 | 36 |
| **Switch to bipolar mode** | 12 | 24 |
| **Change to interleaved stimulation** | 10 | 20 |
| **Use Optistim** | 10 | 20 |
| **Switch to double monopolar mode** | 1 | 2 |

**Table 17: Non-motor adverse effects- select upto 3 top optimization strategies N=50**

|  | **Number of respondents (n)** | **Percentage (%)** |
| --- | --- | --- |
| **Modify amplitude** | 25 | 50 |
| **Modify frequency** | 15 | 30 |
| **Modify pulse width** | 20 | 40 |
| **Activate a segment** | 16 | 32 |
| **Alter electrode location along the lead** | 25 | 50 |
| **Switch to bipolar mode** | 16 | 32 |
| **Change to interleaved stimulation** | 10 | 20 |
| **Use Optistim** | 9 | 18 |
| **Switch to double monopolar mode** | 4 | 8 |

**Table 18: Swallowing related side effects- select upto 3 top optimization strategies N=50**

|  | **Number of respondents (n)** | **Percentage (%)** |
| --- | --- | --- |
| **Modify amplitude** | 30 | 60 |
| **Modify frequency** | 23 | 46 |
| **Modify pulse width** | 18 | 36 |
| **Activate a segment** | 17 | 34 |
| **Alter electrode location along the lead** | 15 | 30 |
| **Switch to bipolar mode** | 16 | 32 |
| **Change to interleaved stimulation** | 6 | 12 |
| **Use Optistim** | 11 | 22 |
| **Switch to double monopolar mode** | 0 | 0 |

**Table 19: Freezing of gait- select upto 3 top optimization strategies N=50**

|  | **Number of respondents (n)** | **Percentage (%)** |
| --- | --- | --- |
| **Modify amplitude** | 17 | 34 |
| **Modify frequency** | 43 | 86 |
| **Modify pulse width** | 19 | 38 |
| **Activate a segment** | 4 | 8 |
| **Alter electrode location along the lead** | 22 | 44 |
| **Switch to bipolar mode** | 6 | 12 |
| **Change to interleaved stimulation** | 21 | 42 |
| **Use Optistim** | 5 | 10 |
| **Switch to double monopolar mode** | 2 | 4 |

**Table 20: What are patients taught at initial programming? (select one or more) N=50**

| **Teaching option** | **Number of respondents (n)** | **Percentage (%)** |
| --- | --- | --- |
| **How to check battery** | 46 | **92%** |
| **How to charge rechargeable devices** | 42 | **84%** |
| **How to turn off the device for procedures** | 38 | **76%** |
| **How to adjust individual parameters** | 23 | **46%** |
| **How to change between groups** | 23 | **46%** |
| **How to mark events on Percept device** | 16 | **32%** |
| **How to put device into MRI mode** | 15 | **30%** |

**Table 21: Following initial programming of DBS STN, when is the medication reduced? N=50**

|  | **Number of respondents (n)** | **Percentage (%)** |
| --- | --- | --- |
| **At the time of the initial programming** | 19 | 38 |
| **One week after initial programming** | 7 | 14 |
| **One month after initial programming** | 12 | 24 |
| **Three months after initial programming** | 5 | 10 |
| **No set time for medication reduction** | 7 | 14 |

**Table 22: Following initial programming of DBS GPi, when is the medication reduced? N=50**

|  | **Number of respondents (n)** | **Percentage (%)** |
| --- | --- | --- |
| **At the time of the initial programming** | 5 | 10 |
| **One week after initial programming** | 8 | 16 |
| **One month after initial programming** | 14 | 28 |
| **Three months after initial programming** | 7 | 14 |
| **No set time for medication reduction** | 16 | 32 |

**Table 23: What do you use to base your decision on medication reduction? (select one or more)**

* **Criteria Used to Base Medication Reduction Decisions (N = 50)**

| **Decision criterion** | **Number of respondents (n)** | **Percentage (%)** |
| --- | --- | --- |
| **Patient perception of improvement** | 34 | **68%** |
| **OFF medication UPDRS score reduction ≥30%** | 29 | **58%** |
| **Patient reported side effects** | 22 | **44%** |
| **Visual assessment of patient response (no formal scale)** | 21 | **42%** |
| **Assessment of BrainSense data** | 15 | **30%** |
| **Patient report of skipping doses** | 5 | **10%** |
| **Other *(e.g.,** | 1 | **2%** |

**Table 24: Rank the factor you use to base your decision on medication reduction N=50**

|  | **Most important %** | **Important %** | **Least important %** |
| --- | --- | --- | --- |
| **Patient perception of improvement** | **46** | **48** | **6** |
| **OFF medication UPDRS score reduction by 30% or more** | **38** | **52** | **10** |
| **Assessment of BrainSense data** | **12** | **56** | **32** |
| **Patient reported side effects** | **42** | **56** | **2** |
| **Patient report of skipping doses** | **10** | **62** | **28** |
| **Visual assessment of patient response demonstrating improvement without a formal scale** | **44** | **44** | **12** |

**Table 25: Do you ask patients to come off dopaminergic medication for subsequent programming sessions? N=50**

|  | **Number of respondents (n)** | **Percentage (%)** |
| --- | --- | --- |
| **Always** | 20 | 40 |
| **Almost always** | 17 | 34 |
| **Sometimes** | 10 | 20 |
| **Never** | 37 | 72 |

**Table 26: What do you recommend for planning patient visits following initial programming? N=50**

|  | **Number of respondents (n)** | **Percentage (%)** |
| --- | --- | --- |
| **Every 2 weeks post initial programming until stimulation is optimized** | 19 | 38 |
| **Every month post initial programming until stimulation is optimized** | 20 | 40 |
| **Every 2 months post initial programming until stimulation is optimized** | 5 | 10 |
| **Every 3 months post initial programming until stimulation is optimized** | 6 | 12 |

**Table 27: Are you currently using a BrainSense sensing-enabled DBS system? N=50**

|  | **Number of respondents (n)** | **Percentage (%)** |
| --- | --- | --- |
| **Yes** | 42 | 84 |
| **No** | 4 | 8 |
| **Plan to use** | 4 | 8 |

**Table 28: When do you initiate BrainSense? N=42**

|  | **Number of respondents (n)** | **Percentage (%)** |
| --- | --- | --- |
| **At initial programming** | 26 | 61.90476 |
| **After the initial programming- at second or subsequent programming sessions** | 11 | 26.19048 |
| **When results of clinical programming are not optimal** | 3 | 7.142857 |
| **Other** | 2 | 4.761905 |

**Table 29: Do you perform a BrainSense Survey in clinic? N= 42**

|  | **Number of respondents (n)** | **Percentage (%)** |
| --- | --- | --- |
| **Yes, always** | 22 | 52.38095 |
| **Most of the time** | 15 | 35.71429 |
| **Occasionally** | 4 | 9.52381 |
| **No, never** | 1 | 2.380952 |

**Q 36 What is the purpose of conducting a BrainSense Survey? (Select one or more)**

**(N = 41)**

Result included in the manuscript-Table 2

**Q37 Does BrainSense influence programming adjustment? (N = 42)**

Result included in the manuscript-Table 3

**Q 38 Which feature of BrainSense influences programming adjustment? (select one or more) ***

**BrainSense Features Influencing Programming Adjustment (N = 40)**

Result included in the manuscript-Table 3

**Q 39 Rank the BrainSense features which influence programming adjustment? N=40 (Rank most to least)**

**Survey> Streaming>Timeline> Events**

**Q 40 Does BrainSense influence medication adjustment? N=42**

Result included in the manuscript-Table 3

**Q 41 Which feature of BrainSense influences medication adjustment? (select one or more)**

**BrainSense Features Influencing Medication Adjustment (N = 35)**

Result included in the manuscript-Table 3

**Table 30:** **Has BrainSense threshold feature aided in identifying whether a patient is over treated or undertreated after initial programming? N= 42**

|  | **Number of respondents (n)** | **Percentage (%)** |
| --- | --- | --- |
| **Yes** | 17 | 40.5 |
| **Occasionally** | 13 | 31.0 |
| **No** | 9 | 21.4 |
| **Not aware of threshold feature** | 3 | 7.1 |

**Table 31: After the launch of Adaptive Deep Brain Stimulation in India, you intend to incorporate it into your clinical practice for treating PD patients? N=50**

|  | **Number of respondents (n)** | **Percentage (%)** |
| --- | --- | --- |
| **Strongly agree** | 28 | 56 |
| **Agree** | 17 | 34 |
| **Neutral** | 5 | 10 |
| **Disagree** | 0 | 0 |
| **Strongly disagree** | 0 | 0 |

**Table 32: Do you foresee Adaptive DBS leading to improved patient outcomes in PD management	N=50**

|  | **Number of respondents (n)** | **Percentage (%)** |
| --- | --- | --- |
| **Definitely** | 24 | 48 |
| **Maybe** | 22 | 44 |
| **Unlikely** | 0 | 0 |
| **Cannot comment at this point** | 4 | 8 |

**Table 33: For suboptimal patient response to DBS, what strategies are used to determine if lead repositioning is required? (select one or more)**

**(N = 50)**

| **Strategy** | **Number of respondents (n)** | **Percentage (%)** |
| --- | --- | --- |
| **Pre‑/post‑operative imaging merge with review of lead location** | 44 | **88%** |
| **Repeat imaging if programming is challenging or patients have suboptimal benefit / side effects** | 40 | **80%** |
| **Repeat monopolar review / initial programming procedure** | 30 | **60%** |
| **Use sensing to optimize results** | 25 | **50%** |
| **Verify with SureTune** | 23 | **46%** |
| **Second opinion by another DBS programming clinician** | 8 | **16%** |
| **Revisit L‑dopa challenge test and PD diagnosis** | 8 | **16%** |
| **Consider replacing current system with sensing when choosing repositioning** | 7 | **14%** |
| **Other** | 0 | **0%** |

**Table 34: For suboptimal patient response to DBS,  rank the strategies you use to determine if lead repositioning is required?  N=50**

|  | **Most likely %** | **Likely %** | **Least likely %** |
| --- | --- | --- | --- |
| **Repeat monopolar review/ initial programming procedure** | **56** | **30** | **14** |
| **Second opinion by another DBS programming clinician** | **6** | **48** | **46** |
| **Pre-/post-operative imaging merge with review of lead location** | **64** | **34** | **2** |
| **Repeat imaging if programming is challenging and patients are not achieving therapeutic benefit or are having side effects** | **64** | **36** | **-** |
| **Use sensing to optimize results** | **32** | **56** | **12** |
| **Verify with SureTune** | **38** | **46** | **16** |
| **Consider replacing current system with sensing if choosing re-positioning** | **4** | **48** | **48** |
| **Revisit L-dopa challenge test and diagnosis of PD** | **8** | **32** | **60** |
